# Supramolecular Maleimide–Styrene Copolymers with Fluorophenyl Side Chains for High-Performance Guest–Host Electro-Optic Materials

**DOI:** 10.1021/prechem.5c00365

**Published:** 2026-02-17

**Authors:** Danning Lyu, Shivani Pathania, Jingdong Luo

**Affiliations:** † Department of Chemistry, 53025 City University of Hong Kong, Hong Kong SAR, China; ‡ Shenzhen Research Institute, City University of Hong Kong, Shenzhen 518057, China

**Keywords:** supramolecular chemistry, nonclassical hydrogen bonding, electro-optic polymers, attenuated total reflection, thermal stability, nanoindentation

## Abstract

This study reports the precise molecular design of maleimide-styrene alternating copolymers, poly­(MI-*alt*-S), as host polymers for supramolecular guest–host electro-optic (EO) materials, combining a benchmark push–pull tetraene chromophore to achieve performance optimization. Leveraging the inherent sequence control, high glass transition temperature, and synthetic versatility of poly­(MI-*alt*-S), four fluorophenyl-substituted copolymers were synthesized to incorporate three to five fluorine atoms onto the phenyl side chains, tuning the weak hydrogen bonding interactions. Guided by theoretical calculations, this work encompasses monomer synthesis, copolymerization, and spectroscopic characterization of fluorobenzyl ether-functionalized poly­(MI-*alt*-S) polymers to reveal how subtle variations in fluorine substitution govern nanoscale morphology, critical cracking thickness, electric field poling, and EO performance of guest–host polymers. EO activity, polar order, and thermal stability of poled films were evaluated using high-accuracy attenuated total reflection (ATR) measurement. Notably, two of the polymers demonstrated large and thermally stable EO coefficients of ∼170 pm/V at 1306 nm and ∼100 pm/V at 1541 nm, among the best ATR-validated performances for guest–host polymers. These findings highlight that tailored nonclassical hydrogen bonds from fluorophenyl side chains effectively balance intermolecular interactions, mechanical properties, orientational polarization, and thermal stability, illustrating how molecular-level precision in the design of guest–host EO polymers translates into optimized photonic functionality.

## Graphical abstract

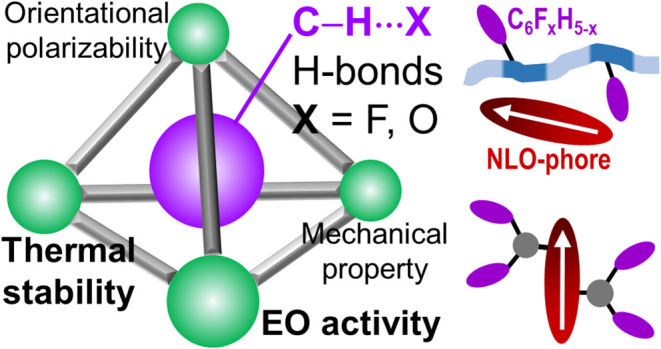

## Introduction

Over the past two decades, the rapid development of high-performance organic electro-optic (EO) materials and modulators has mirrored advances in global communication infrastructures, cloud services, and AI workloads.^1–9^ This progress drives the demand for scalable, power-efficient, and long-reach optical interconnect solutions. As a general strategy, the design and synthesis of highly efficient organic EO (OEO) materials utilize multifluorinated aromatics to introduce favorable interaction energetics and a site isolation effect around highly polarizable π-conjugated chromophores, thereby enhancing and stabilizing the electric-field-induced polar order in dendronized chromophores, multiarm dendrimers, and dendronized side-chain polymers.^2,3,9^ Partial fluorination also enhances the solution processability, optical transparency, and dielectric properties of OEO materials, making them promising for device fabrication and integration.^10–18^ Given that research on OEO materials embodies a unique and rapidly advancing platform in supramolecular chemistry and molecular photonics, this work aims at addressing the key challenge in implementing the latest research progress in precision supramolecular engineering of low glass transition temperature (*T*_g_) EO dendrimers to the rational design and synthesis of scalable high-*T*_g_ guest–host EO polymers.

Phenyl–perfluorophenyl π–π stacking, with a cohesive energy ranging from 20 to 25 kJ/mol per phenyl ring,^19–21^ generally improves the temporal stability of OEO dendrimers but gives mixed results for polar order due to the negative entropy, as shown by **Type I** and **Type II** dendrimers in Figure 1.^10,11,22^ By contrast, introducing a single or double F-to-H substitution on the perfluorophenyl ring can change the self-assembly of fluoroaromatics from π–stacking-dominated interactions to weaker, C–H···X (X = F and O) hydrogen-bonding interactions.^23–25^ This modification promotes acentric hydrogen bond (H-bond) formation while reducing centrosymmetric π–π stacking and electrostatic interactions, as demonstrated in a recent study of **Type III** dendrimers (Figure 1).^25^ The interaction energy of these nonclassical H-bonds does not exceed 10 kJ/mol, and the C–H···X contacts are dispersive, flexible, and entropically stabilized.^26^ Consequently, the new H-bonded fluorophenyl motif reconciles two fundamental but conflicting requirements: the high rotational freedom of push–pull tetraene chromophores (PPT-phores) during poling and the temporal stability of aligned PPT-phores against thermal relaxation during operation. This entropy-driven approach therefore enables a new design strategy for developing highly efficient and thermally stable OEO materials.^25^

**Figure 1:**
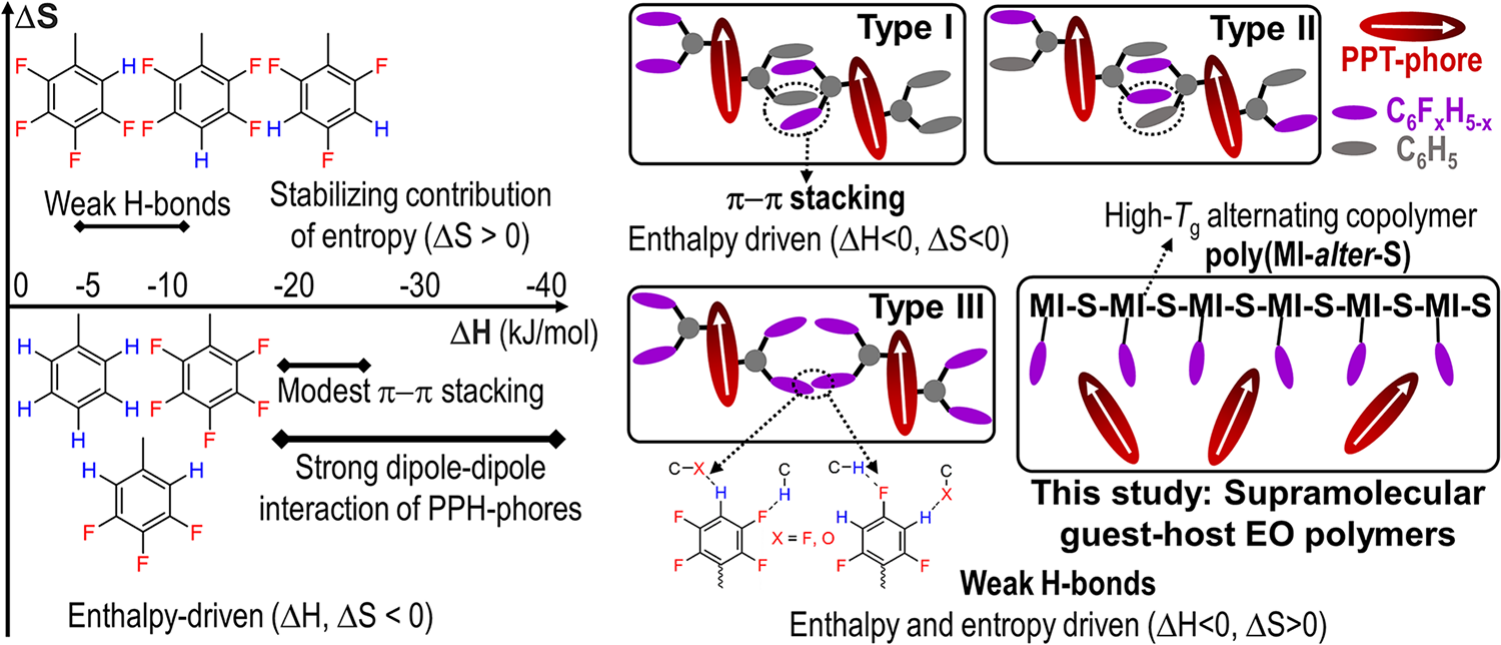
**Left**: Ranges of interaction enthalpy (Δ*H*) and entropy (Δ*S*) for noncovalent interactions span weak hydrogen bonding, modest π–π stacking in toluene and fluorinated analogues, and strong dipole–dipole interactions in push–pull tetraene chromophores (PPT-phores). The atoms with significant partial atomic charges, marked in blue for positive and red for negative, are shown as descriptors of molecular interactions. **Right:** Precision design and synthesis of organic EO dendrimers incorporating supramolecular fluorophenyls to the donor and π-bridge of PPT-phores. **Type I** dendrimers incorporate symmetric dendrons for phenyl-perfluorophenyl π–π stacking interactions; **Type II** dendrimers incorporate asymmetric *co*-dendrons for phenyl-perfluorophenyl π–π stacking or H-bond interactions; **Type III** dendrimers incorporate a singular fluorophenyl synthon for weak H-bonds. In this study, a high-*T*_g_ alternating copolymer, poly­(MI-*alt*-S), featuring a singular fluorophenyl side chain, is presented to facilitate the synthesis and comprehensive evaluation of supramolecular guest–host EO polymers..

In this study, we select maleimide-styrene alternating copolymers, poly­(MI-*alt*-S), as the host polymer and a benchmark dipolar PPT-phore as the guest to formulate new guest–host EO polymers. The poly­(MI-*alt*-S) offers a high *T*_g_ and versatile side-chain modifications, which have previously supported the synthesis of side-chain and cross-linkable EO polymers.^27–30^ However, until very recently, its potential as a host polymer in guest–host EO materials has not been systematically explored. Herein lies an important opportunity for guest–host polymers as a straightforward and reproducible platform for investigating supramolecular interactions and optimizing multiple properties of OEO materials.

Therefore, building on prior work with OEO dendrimers, this study introduces a series of fluorophenyl-substituted poly­(MI-*alt*-S) copolymers containing three to five fluorine atoms on the phenyl ring to fine-tune supramolecular interactions that govern structure–property relationships in the formulated guest–host systems. In addition, the attenuated total reflection (ATR) technique is employed as a standard and accurate measurement method for evaluating the EO efficiency and thermal stability of poled polymer slab waveguide films, allowing reliable comparison, interpretation, and utilization of EO results.^31,32^

In the following, we report the theoretical calculations, monomer synthesis, copolymerization, and spectroscopic characterization of new poly­(MI-*alt*-S) polymers with fluorobenzyl ether side chains. We further study the morphology, critical cracking thickness, electric field poling, EO activity, and thermal stability of guest–host EO polymers. Notably, two formulated materials exhibit large and thermally stable EO coefficients of ∼170 pm/V at 1306 nm and ∼100 pm/V at 1541 nm, among the best performance to date for guest–host EO polymers verified by the high-precision ATR technique. The study demonstrates that nonclassical H-bonds from fluorophenyl side chains balance intermolecular interactions, elastic modulus, hardness, orientational polarization, and thermal stability, thereby optimizing the key properties of EO polymers for photonic applications.

## Results and Discussion

### Calculation of the Partial Atomic Charges of *N*-Phenylmaleimide Monomers with Fluorobenzyl Ether Groups

We began by using density functional theory (DFT) to calculate the partial atomic charges and dipole moments of *N*-phenylmaleimido monomers with fluorobenzyl ether groups, where the Charge Model 5 (CM5) values serve as a basic and accurate descriptor for molecular interactions (Figures 2 and S1).^33^ Typically, C–H···O bonds originate from interactions between a soft donor (C–H) and a soft acceptor (O), whereas C–H···F bonds form with a hard C–F acceptor and a C–H donor with the increasing acidity and hardness.^23,24^ The *N*-phenylmaleimide moiety is distinguished by a highly negative charge at its two carbonyl oxygens, while the two olefinic hydrogens of the imide moiety show the highest positive charge, and the hydrogens of the phenyl ring are comparatively less positively charged. The substituents of fluorobenzyl ether groups introduce more electronegative fluorine and oxygen atoms into the structure, altering the dipole moments of the monomers. These parameters are essential for understanding the formation of extended supramolecular interactions within the model compounds of the monomers and the resulting poly­(MI-*alt*-S) polymers.

**Figure 2:**
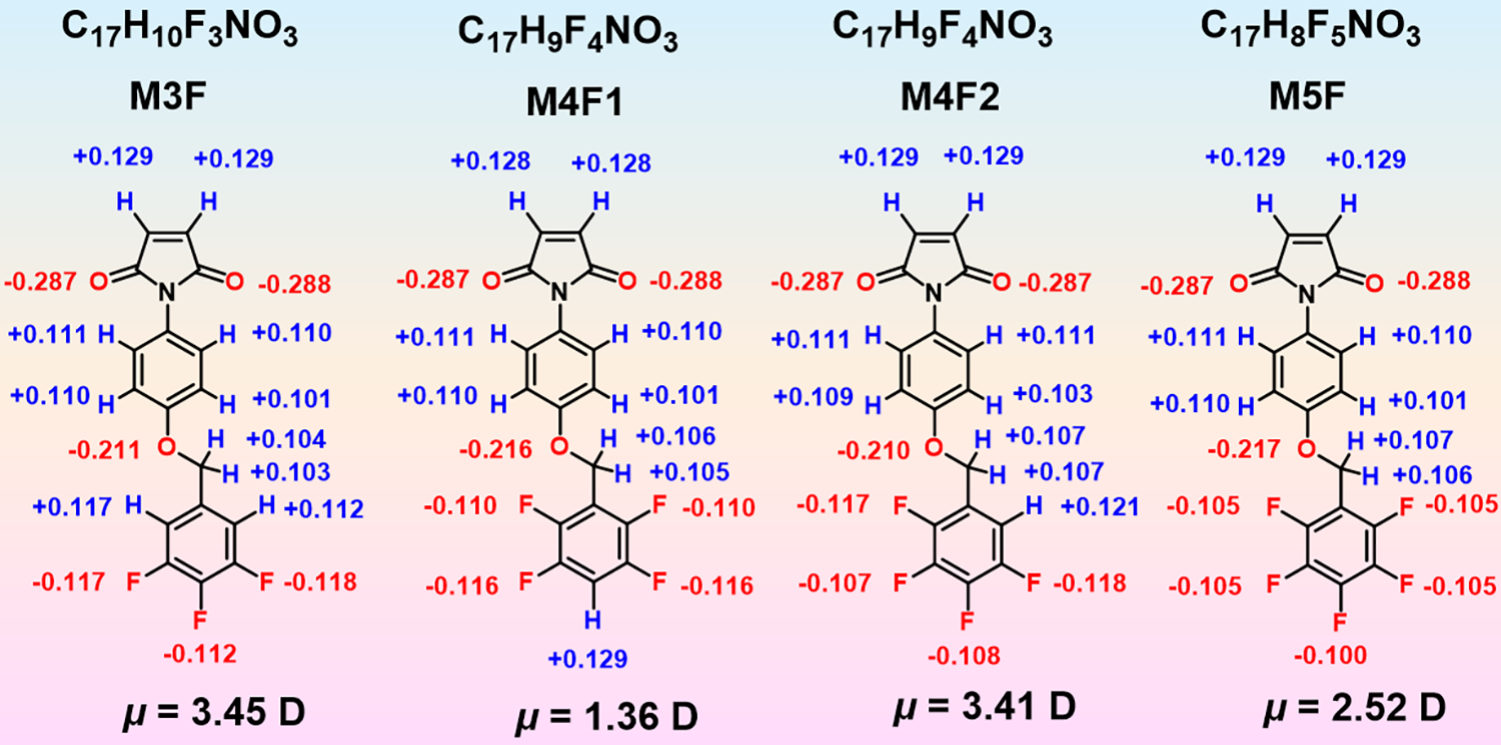
Dipole moment (μ) and partial atomic charges, marked in blue for the positive and red for the negative, were calculated in the gas phase using the Charge Model 5 (CM5) by density functional theory (DFT). These calculations were performed using Gaussian 16 at the B3LYP/6-31G+(d,p) level, providing a quantitative description of molecular interactions.

### Synthesis and Structural Characterization

The synthesis of poly­(MI-*alt*-S) copolymers is shown in Scheme 1, with detailed synthetic procedures and chemical characterizations provided in the Supporting Information. Initially, the precursor *N*-(4-hydroxyphenyl)­maleimide was protected with furan via a Diels–Alder [4 + 2] cyclo-addition reaction to produce compound **1**. This was followed by a Mitsunobu etherification reaction in the presence of diisopropyl azodicarboxylate (DIAD) and triphenylphosphine at ambient temperature.^34^ The resultant compounds **2–5** were then deprotected at elevated temperature by the retro-Diels–Alder reaction, yielding the key intermediates of *N*-phenylmaleimido monomers with fluorobenzyl ether groups, specifically **M3F**, **M4F1**, **M4F2**, and **M5F**. Finally, radical polymerization of the equimolar mixture of the maleimido monomer and comonomer α-methylstyrene was conducted using 2,2′-azobis­(2-methylpropionitrile) (AIBN) as the initiator, producing the poly­(MI-*alt*-S) copolymers, namely **P3F**, **P4F1**, **P4F2**, and **P5F**, respectively.^27–30^ Overall, the reactions were carried out under standard conditions with good yields.

**Scheme 1:**
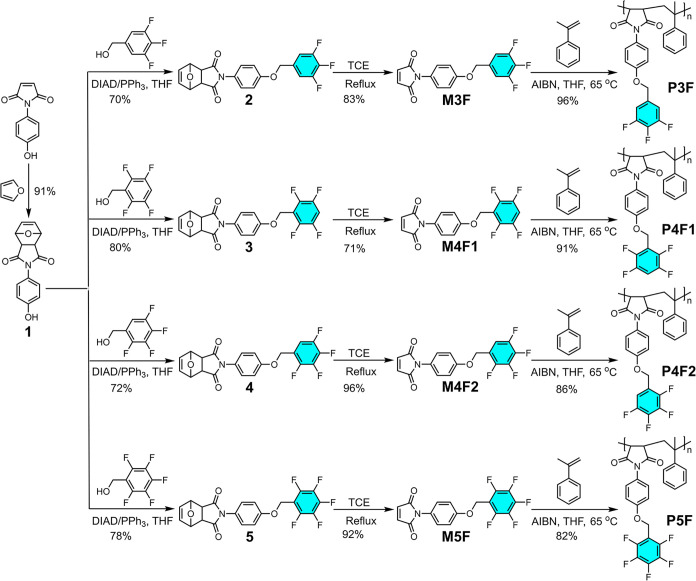
Synthesis of the Maleimido Monomers and Copolymers with Fluorobenzyl Ether Groups^a^

a DIAD, diisopropyl azodicarboxylate; TCE, 1,1,2-trichloroethane; AIBN, 2,2′-azobis­(2-methylpropionitrile).

The structures of the maleimido monomers bearing fluorobenzyl ether groups were confirmed by ^1^H NMR, ^13^C NMR, ^19^F NMR, and HRMS analyses. Based on the relative integration of the benzylic proton signals at approximately 5.10 ppm in the ^1^H NMR spectra, the molar ratio of maleimido to α-methylstyrene units was determined to be 1.0 for all the polymer products, consistent with the feed ratio of comonomers used in the polymerization. From the gel-permeation chromatography (GPC), the weight-averaged molecular weights (*M*_w_) and polydispersity indices (*Đ*, i.e., *M*_w_/*M*_n_) were estimated to be approximately 30.0 kDa/2.8 (*M*_w_/*Đ*) for **P4F1** and **P5F**, 18.0 kDa/2.3 for **P4F2**, and 9.0 kDa/2.2 for **P3F**, respectively (Table S2). Thermal analysis by differential scanning calorimetry (DSC) revealed relatively high *T*_g_s for **P4F1** and **P5F**, significantly higher than those of **P3F** and **P4F2**. Interestingly, the trends in the changes of *M*_w_s and *T*_g_s reverse that of dipole moments for the maleimido monomers (Figure 2). It suggests that the copolymerization kinetics for **P4F1** and **P5F** follow the alternating structure of poly­(MI-*alt*-S) as expected. However, for **P3F** and **P4F2**, the copolymerization kinetics may be affected to have compositional drift in the formed copolymer products, which contain random (not alternating) copolymer structures.^35^ This could be related to the strong dipole–dipole interaction of the maleimido monomers **M3F** and **M4F2**, which can interfere with their copolymerization kinetics with α-methylstyrene.

### Crystal Structures and H-Bonds in **M4F1** and **M4F2**

We obtained quality crystals of **M4F1** and **M4F2** for X-ray analysis (Figure 3). To identify the H-bonds in the crystal structures, we followed Desiraju’s convention, using a H···X distance (*d*) cutoff of 2.80 Å and an angle (θ) greater than 120° to show the close C–H···X (X = F, O) contacts.^23–25^ Also, we compared the crystal structures with that of simple *N*-phenylmaleimide (**NPM**) previously reported.^36^

**Figure 3:**
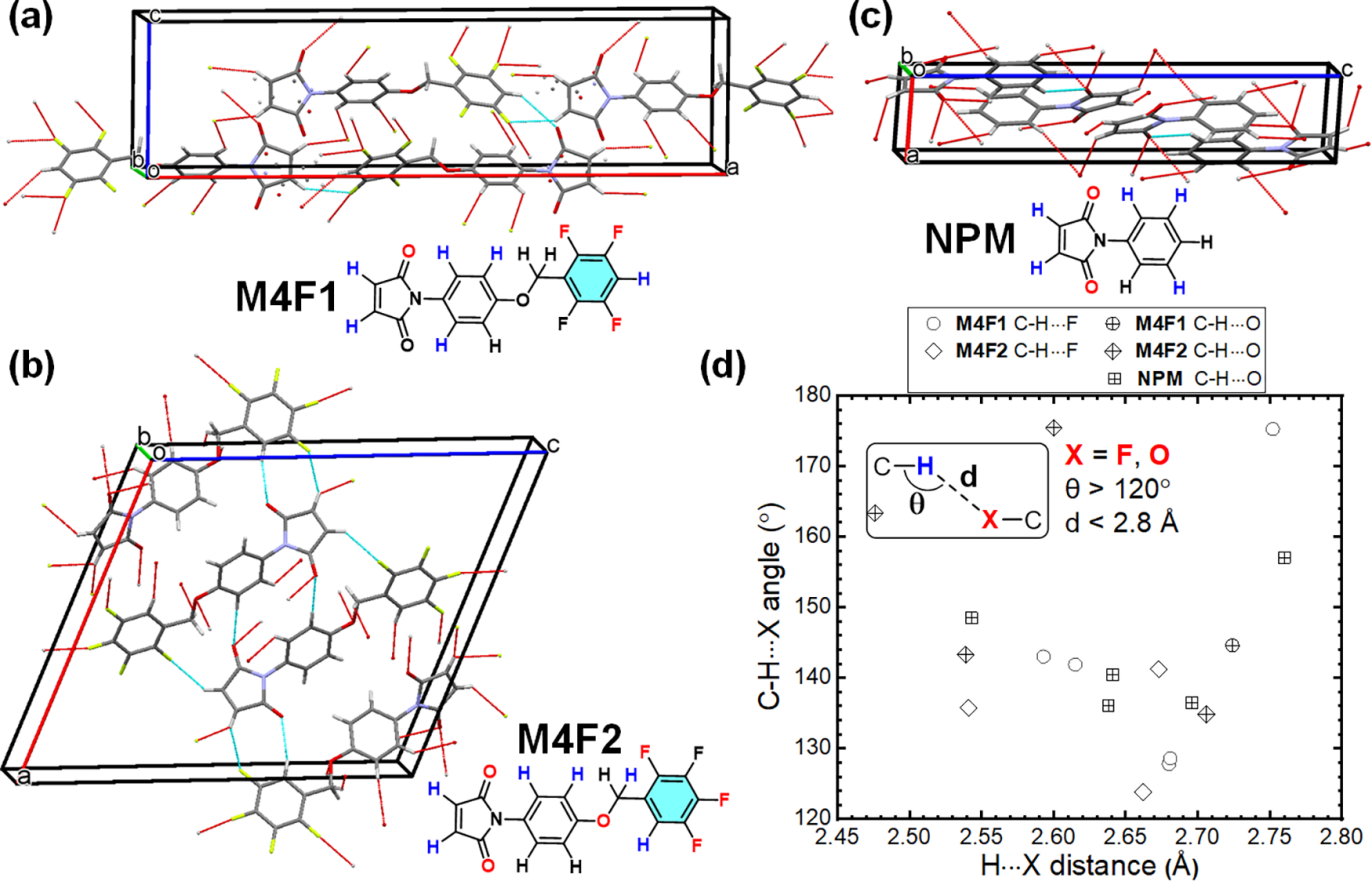
(a) Crystal unit cell and hydrogen bonding of **M4F1**, compared with those from **M4F2** and **NPM** in (b) and (c). The close C–H···X (X = F, O) contacts are indicated by the dotted red or green lines. The structures of three maleimido molecules are shown with hydrogen marked in blue and F/O in red for the identified C–H···X (X = F, O) bonds. (d) Scatterplots of H···X distances versus C–H···X angles for comparing different C–H···X (X = F, O) interactions in these molecules. Inserted: the cutoff C–H···X angle and H···X length were used to define the H-bonds by the Mercury software.

Compound **M4F1** crystallizes in the orthorhombic space group *Pna*2_1_ with disordered maleimido rings in two different orientations (Figure 3a and Table S1). This polar space group with a 2_1_ screw axis organizes the **M4F1** monomers into H-bonded polar chains along the *c*-direction. While no significant π–π interactions are observed, the dipole–dipole interactions appear weak due to the small dipole moment and loose packing of **M4F1**. Interestingly, two C–N bond lengths differ by 0.06 Å, indicating asymmetric polarization of the maleimido group perpendicular to its primary dipole. The spontaneous polarization of the **M4F1** crystal results from the noncentrosymmetric arrangement of the 4-((2,3,5,6-tetrafluorobenzyl)­oxy)­phenyl moiety and the maleimido moiety with asymmetric C–N–C polarization, which is stabilized by extended H-bond interactions involving five C–H···F and one C–H···O contacts.

In contrast, compound **M4F2**, the constitution isomer of **M4F1**, exhibits a larger dipole moment and crystallizes in the centrosymmetric monoclinic space group *P*2_1_/*n*, identical to **NPM** (Figure 3b,c).^36^ The crystal packing of **M4F2** is dominated by π–π stacking interactions, with a plane-to-plane distance of 3.826 Å along the *b*-axis, as well as antiparallel dipole–dipole electrostatic interactions and extended H-bond interactions through three C–H···F and four C–H···O contacts. Unlike **M4F1**, the two C–N bonds in **M4F2** and **NPM** are highly symmetrical. Comparing bond angles and distances of various C–H···X (X = F and O) contacts in these three compounds illustrates that C–H···F interactions are analogous to C–H···O interactions. It suggests that versatile C–H···X synthons (X = F and O) can be developed for the supramolecular engineering of maleimide-containing EO polymers.

### Morphological and Nanoindentation Studies of Guest–Host EO Polymers

Following the standard solution processing protocol, the poly­(MI-*alt*-S) copolymers, namely **P3F**, **P4F1**, **P4F2**, and **P5F**, were mixed with an efficient push–pull tetraene chromophore **AJLZ53**, to formulate four corresponding guest–host polymers, **P3F-AJLZ53**, **P4F1-AJLZ53**, **P4F2-AJLZ53**, and **P5F-AJLZ53**, respectively. Here, **AJLZ53** is a benchmark PPT-phore exhibiting large molecular hyperpolarizability up to 8000 × 10^–27^ esu at 1.3 μm in poled guest–host polymers. The chromophore content in these guest–host polymers was set at loading densities of 27 and 35 wt %, consistent with the benchmark guest–host EO polymer formulated by doping the **AJLZ53** into polycarbonate (referred to as **PC-AJLZ53**) for comparative studies of EO properties.^4–8,37^

Simple poly­(MI-*alt*-S) copolymers are stiff, high-*T*_g_ materials that increase modulus and heat resistance but tend to be relatively brittle on their own. For the fabrication of thin-film waveguides where stress-crack resistance is critical, adding the guest chromophore at a high loading density and attaching the supramolecular fluorobenzyl ether side-chain groups can potentially improve the ductility and mechanical properties of guest–host polymers. Scanning electron microscopy (SEM) images showed that the **P4F1-AJLZ53** and **P5F-AJLZ53** films exhibited highly uniform and continuous surface morphology, indicating excellent guest–host compatibility (Figure 4). Specifically, the **P4F1-AJLZ53** films demonstrated small surface roughness, no visible defects, and excellent adhesion to the substrate. Although the **P5F-AJLZ53** films were overall continuous and uniform, a small number of granular precipitates were observed on their surface, likely due to the simple film preparation process in a nonclean-room environment. In sharp contrast, the **P3F-AJLZ53** and **P4F2-AJLZ53** films displayed noticeable surface cracking: the former showed finer and more densely distributed microcracks, while the latter exhibited a relatively uniform large-area fragmentation morphology (Figures 4a–c, and S2).

**Figure 4:**
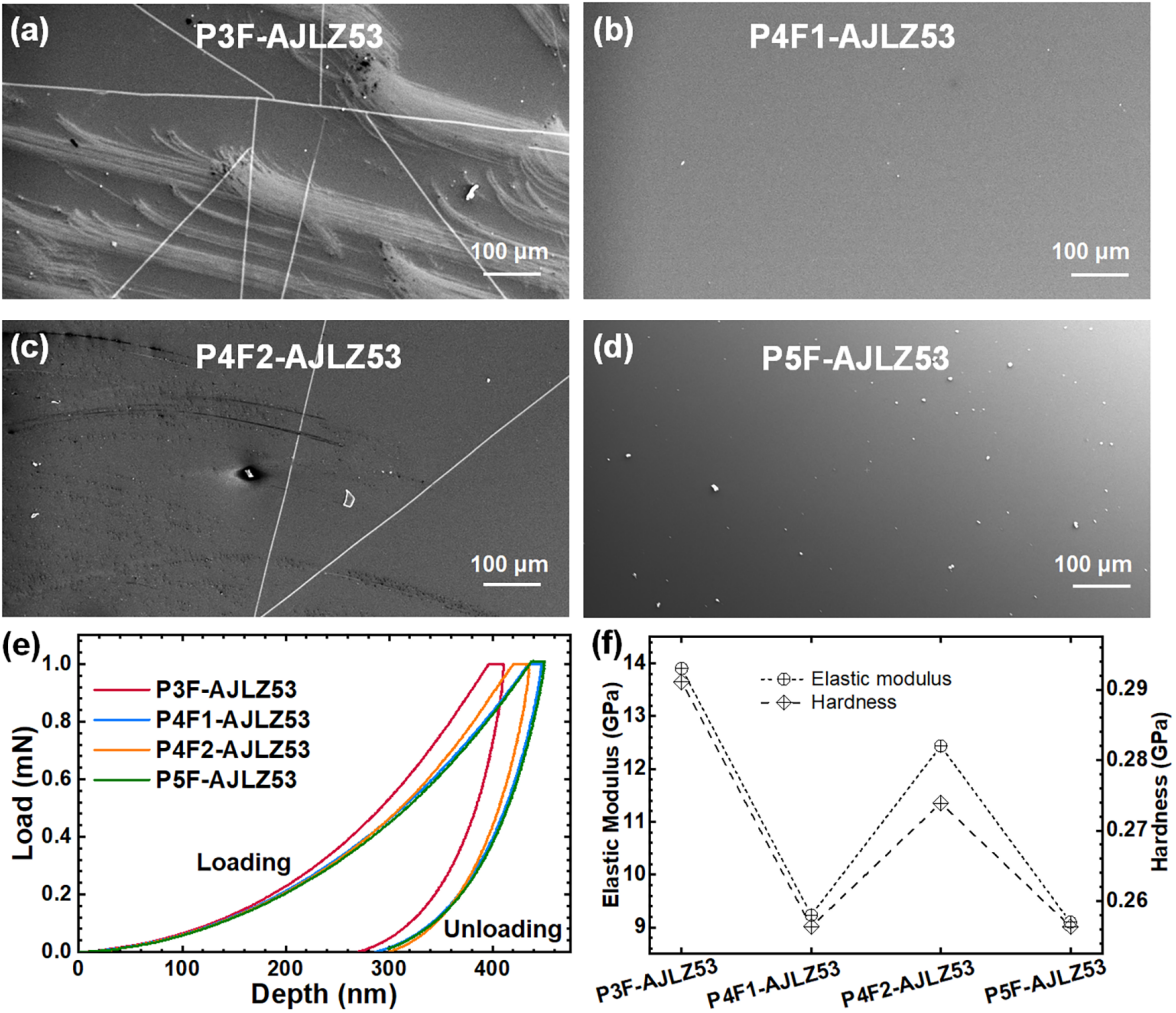
Nanoscale characterization of guest–host polymer films of **P3F-AJLZ53**, **P4F1-AJLZ53**, **P4F2-AJLZ53**, and **P5F-AJLZ53** by scanning electron microscopy (SEM) to evaluate the surface morphology and crack formation of films (a–d), and by nanoindentation tests: (e) load–displacement curves, and (f) elastic modulus and hardness.

Nanoindentation tests were conducted to quantitatively characterize the mechanical properties of these guest–host polymers.^38^ The slope of the load–displacement curves corresponds to the stiffness of the films, in which a steeper slope indicates higher resistance to deformation and thus higher structural rigidity. The **P3F-AJLZ53** exhibits the steepest loading curve, indicating the highest stiffness among the series, while the nearly overlapping curves of **P4F1-AJLZ53** and **P5F-AJLZ53** suggest similar stiffness for these two materials (Figure 4e). During unloading, **P3F-AJLZ53** also displays the lowest residual indentation depth, while the other three systems retain slightly larger depths, reflecting less elastic recovery capabilities for these materials. The elastic modulus and hardness values of these guest–host polymers follow the ranking of **P3F-AJLZ53** > **P4F2-AJLZ53** > **P4F1-AJLZ53** ≈ **P5F-AJLZ53**, matching the corresponding dipole moments of the maleimido monomers (Figures 2 and 4f). Within this series, the nanoindentation-derived modulus and hardness serve as comparative indicators rather than absolute, stand-alone quantities, helping to relate subtle structural variations in the guest–host polymers to corresponding changes in mechanical properties.

The X-ray data of **M4F1** and **M4F2** reveal different supramolecular interactions based on different fluorobenzyl-ether-attached maleimido monomers in ordered crystalline forms. It is anticipated that when these weakly interacting synthon structures are incorporated into poly­(MI-*alt*-S) copolymers **P4F1** and **P4F2**, they might alter their interaction energetics. This could significantly impact the mechanical properties of solid films. In **M4F1** and **P4F1**, the dominant C–H···F contacts contribute significantly to entropy stabilization, resulting not just from the mass of the fluorine atom but also its deformability.^25,26^ These packing motifs guide the polymer chain to self-assemble into a uniform, three-dimensional network with well-distributed internal stress. The network is supported by numerous flexible C–H···F contacts, which act as cooperatively responsive “ductile units”. These dynamic, weak interactions dissipate localized stress efficiently across the films through vibration and deformation, thereby mitigating stress concentration and increasing the critical cracking thicknesses of drying polymer films.

In contrast, **M4F2**′s asymmetric fluorination and larger dipole moment lead to an anisotropic, denser packing mode, with more C–H···O contacts forming stronger and more rigid H-bonds than those in **M4F1**. While this may create energetically stable local assemblies, it restricts the formation of a long-range, uniform three-dimensional superstructure, thereby affecting the mechanical ductility and macroscopic homogeneity of the resultant **P4F2**.

### Electric Field Poling and EO Property Study for Guest–Host Polymers

The **P4F1-AJLZ53** and **P5F-AJLZ53**, two guest–host systems exhibiting high optical quality and excellent mechanical properties, were selected to conduct electric field poling and ATR measurements. The **P4F1-AJLZ53** and **P5F-AJLZ53** films showed strong near-infrared (NIR) optical absorption from the chromophore with the maximum wavelength (λ_max_) at ∼850 nm, similar to that of **PC-AJLZ53**. The higher absorption from the low-energy shoulder peak at ∼950 nm indicates a higher polarity of **P4F1** and **P5F** than that of **PC** (Figure S3).

The guest–host films were contact-poled under an electric field strength of ∼90 V/μm, near the materials’ *T*_g_s, while monitoring leakage currents using a Source Meter Unit to prevent dielectric breakdown. The refractive indices *n*_TE_ and *n*_TM_, respectively in TE (transverse electric) and TM (transverse magnetic) polarization, and EO coefficients (i.e., *r*_13_ and *r*_33_ values) of poled guest–host films are determined in slab waveguides on a prism-coupler system Metricon 2010/M by the ATR technique at the wavelengths of 1306 and 1541 nm.^31,32^ The Metricon 2010/M measures the reflectivity and mode angles of slab waveguides, notably without the lock-in amplifier (LIA), for the rapid and traceable characterization of thin-film refractive indices and EO coefficients (Figures S4–S6). Its high accuracy has been verified by commercial thin-film lithium niobate and benchmark EO polymers.

From the modulated ATR spectra of poled films, the *r*-coefficients of EO films are given by Taylor expansion to the first order of linear approximation:

1 $$r_{13}=\frac{2d}{n_{\mathrm{TE}}^{3}V_{\mathrm{mod}}}\Delta n_{\mathrm{TE}}=\frac{2d}{n_{\mathrm{TE}}^{3}V_{\mathrm{mod}}}\Delta R_{\mathrm{TE}}/\left(\frac{\partial R_{\mathrm{TE}}}{\partial N_{\mathrm{eff}}^{s}}\frac{\partial N_{\mathrm{eff}}^{s}}{{\partial n}_{\mathrm{TE}}}\right)$$

2 $$r_{33}=\frac{2d}{n_{\mathrm{TM}}^{3}V_{\mathrm{mod}}}\left[\Delta R_{\mathrm{TM}}+\frac{\partial R_{\mathrm{TM}}}{\partial N_{\mathrm{eff}}^{p}}\frac{\partial N_{\mathrm{eff}}^{p}}{{\partial n}_{\mathrm{TE}}}\frac{n_{\mathrm{TE}}^{3}r_{13}V_{\mathrm{mod}}}{2d}\right]/\left(\frac{\partial R_{\mathrm{TM}}}{\partial N_{\mathrm{eff}}^{p}}\frac{\partial N_{\mathrm{eff}}^{p}}{{\partial n}_{\mathrm{TM}}}\right)$$

where *d* is the film thickness, *V*_mod_ is the modulation voltage of direct current (DC) or alternating current (AC), *R*_TE_ and *R*_TM_ are the measured reflectivity in ATR spectra with the changes of effective refractive indices *N*_eff_^*s*^ for *s*- and *N*_eff_^*p*^ for *p*-polarized waves, and Δ*n*_TE_ and Δ*n*_TM_ are the changes in refractive indices modulated by the EO effect, respectively in TE and TM modes. The ∂*R*/∂*N* derivatives are inherent in the ATR spectra, while the ∂*N*/∂*n* terms are basically 1.0 for multimode waveguides, except *∂N*_eff_^*p*^/*∂n*_TE_ ≪ *∂N*_eff_^*p*^/*∂n*_TM_ (thus the second term in eq 2 can be ignored).^32^

The poled guest–host films exhibited a large poling-induced positive optical birefringence, Δ*n*, defined as the index difference between *n*_TM_ and *n*_TE_ (Table 1). The optical anisotropy of poled EO films is strongly dependent on both the chromophore loading and host polymers, indicating different degrees of poling-induced polar order of guest–host films. With equivalent chromophore loading, the poled films of **P4F1-AJLZ53** give a larger Δ*n* and a higher order parameter (Φ) compared to those of**PF5-AJLZ53** and **PC-AJLZ53**. This suggests that a single F-to-H substitution from **M4F1** to **M5F** has a considerable impact on the poling efficiency of guest–host polymers.^25,26^ In particular, the Φ of poled **P4F1-AJLZ53**-35% is up to 0.160 as one of the highest order parameters for OEO materials.

**Table 1:** Comparison of the Optical Absorption, Poling Condition, Refractive Indices, and EO Coefficients by ATR for the Poled Film of Guest-Host Polymers

Polymer formulation (Thickness)	λ_max_ ^a^ (nm)	Poling condition, *T* (°C), *E* (V/μm)	*n*_TE_/*n*_TM_ at 1306 nm	*n*_TE_/*n*_TM_ at 1541 nm	Φ^b^	*r*_13_/*r*_33_ at 1306 nm (pm/V)	*r*_13_/*r*_33_ at 1541 nm (pm/V)
**P4F1-AJLZ53**-27% (1.844 μm)	849 (953)	136, 92	1.6893/1.7886	1.6592/1.7062	0.099	34.1/131.0	24.2/71.2
**P5F-AJLZ53**-27% (1.707 μm)	839 (957)	142, 91	1.6810/1.7725	-	0.091	31.4/112.1	-
**P4F1-AJLZ53**-35% (2.287 μm)	852 (959)	131, 90	1.7317/1.8742^c^	1.6882/1.7830^c^	**0.160** ^c^	51.9/177.5^c^	37.0/101.7^c^
**P5F-AJLZ53**-35% (1.485 μm)	837 (956)	139, 90	1.7261/1.8637	1.6839/1.7475	0.121	46.6/159.0	32.9/93.8
**PC-AJLZ53**-35% (2.526 μm)	835 (956)	130, 90	1.7158/1.8408	1.6779/1.7630	0.154	56.0/169.2	38.0/106.5

a The maximum wavelengths with the shoulder peak shown in parentheses.

b The order parameters of poled films were calculated from the poling-induced optical birefringence.

c Highest birefringent indices, order parameter, and EO coefficients are shown in bold. The reported *r*-coefficients presented as decimal numbers do not indicate the absolute measurement errors, but rather directly represent the analytical results obtained from ATR measurements.

Additionally, it is important to note that the relative errors in determining refractive index, thickness, and *r*-coefficients were estimated to be less than 10% in multimode waveguide models. The uncertainties in *r*-coefficients measured by the ATR method are significantly smaller than those obtained using the Teng–Man simple reflection technique.^39,40^ The Teng-Man method tends to substantially inflate the *r*-coefficients of poled, highly birefringent EO films.^31,32,39,40^ Consequently, contingent Teng-Man results must undergo rigorous corrections using the optical anisotropic model and quantitative multiple-reflection analysis before being compared to the ATR results.

Based on the series of LIA-free ATR measurements, the *r*-coefficients of these guest–host polymers were calculated using eqs 1 and 2 (Table 1), and some spectral processing was illustrated in Figures 5 and 6. Herein the LIA-free ATR technique uses the commercial prism coupler and function generator with standard operation procedures to provide rapid and reliable measurements of thin-film EO coefficients at low to modest modulation frequencies. It allows us to collect a large amount of modulated ATR spectra for the study, notably without using the complex system-design platforms. Equally important, the thermal stability of poled guest–host films of **P4F1-AJLZ53-**35% and **P5F-AJLZ53-**35% was examined at 85 °C for 500 h and compared to the **PC-AJLZ53-**35%.

**Figure 5:**
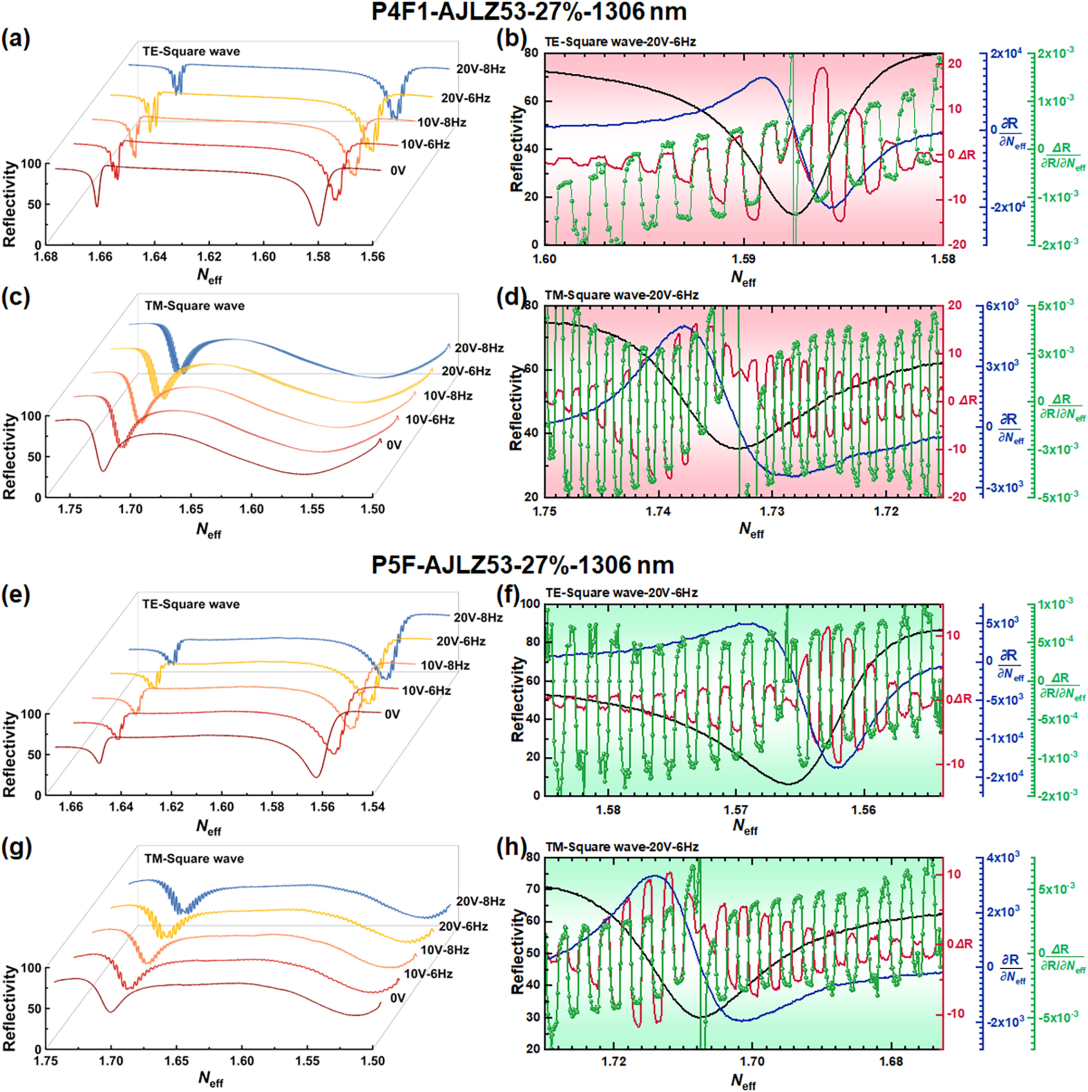
Lock-in free ATR measurement and data processing for EO properties of **P4F1-AJLZ53**-27% at 1306 nm: (a) In TE mode, the reflectivity as a function of the effective refractive index (*N*_eff_), unmodulated (0 V) and modulated by square-wave voltages of different frequencies and different voltages. (b) The reflectivity (*R*), modulation-induced reflectivity change (Δ*R*), first derivative (∂*R*/∂*N*_eff_), and Δ*R*/(∂*R*/∂*N*_eff_) versus *N*_eff_ under a 20 V square-wave voltage at 6 Hz for TE mode. (c) In TM mode, the reflectivity as a function of the *N*_eff_, unmodulated (0 V) and modulated by square-wave voltages of different frequencies and different voltages. (d) Δ*R*, ∂*R*/∂*N*_eff_, and Δ*R*/(∂*R*/∂*N*_eff_) versus *N*_eff_ under a 20 V square-wave voltage at 6 Hz for TM mode. (e–h) For **P5F-AJLZ53**-27%, the ATR measurements and calculation procedures were performed following the same analytical sequence (a–d) as those used for **P4F1-AJLZ53**-27%. The calculated Δ*R*/(∂*R*/∂*N*_eff_) from the ATR measurements is the refractive index change in poled films induced by the Pockels effect.

**Figure 6:**
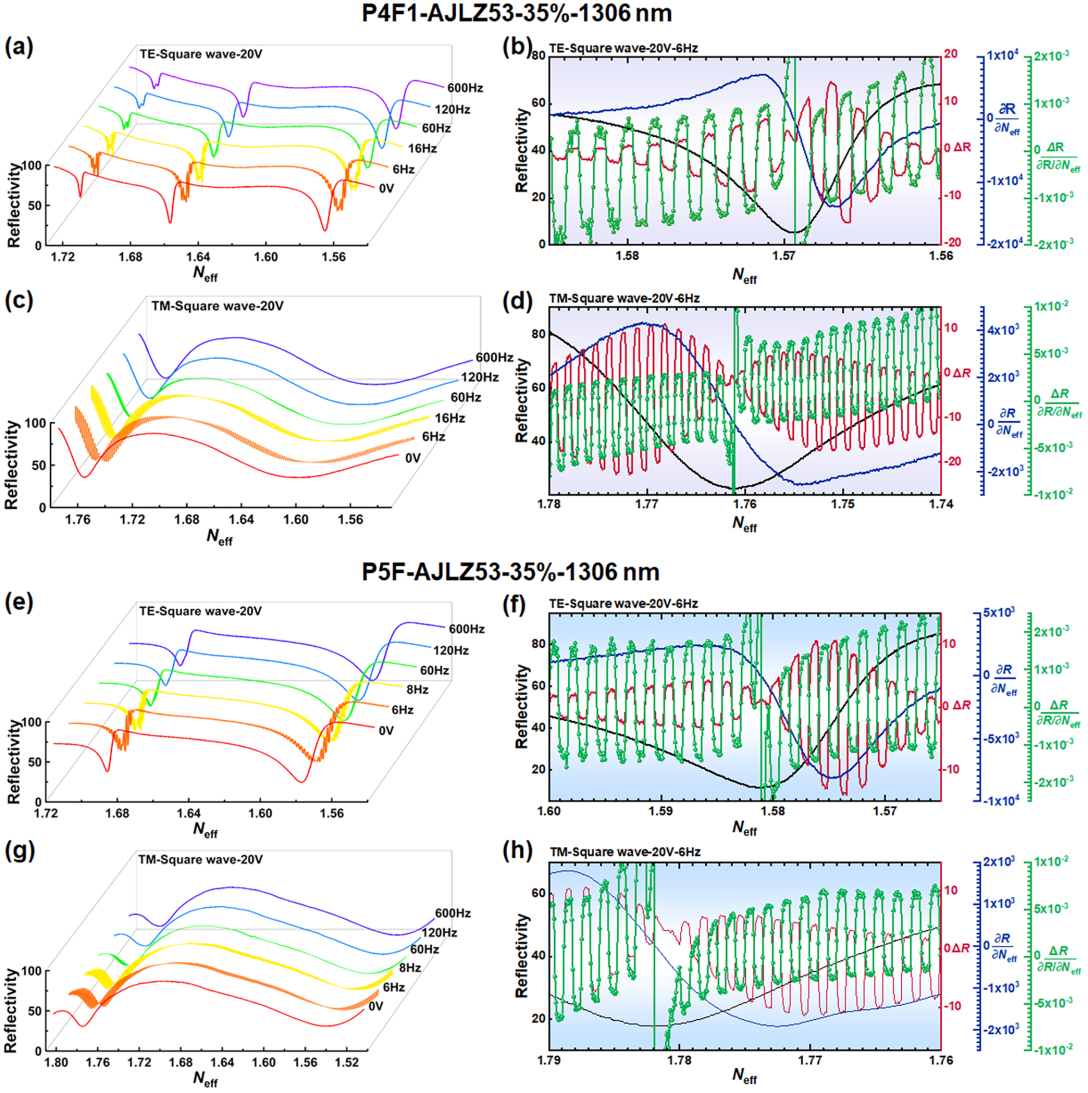
Lock-in free ATR measurement and data processing for the **P4F1-AJLZ53**-35% at 1306 nm: (a) In TE mode, the reflectivity as a function of the effective refractive index (*N*_eff_), unmodulated (0 V) and modulated by a square-wave voltage at different frequencies (20 V). (b) The reflectivity (Δ*R*), its first derivative (∂*R*/∂*N*_eff_), and Δ*R*/(∂*R*/∂*N*_eff_) versus *N*_eff_ under a 20 V square-wave voltage at 6 Hz for TE mode. (c) In TM mode, the reflectivity as a function of *N*_eff_, unmodulated (0 V) and modulated by a square-wave voltage at different frequencies (20 V). (d) Δ*R*, ∂*R*/∂*N*_eff_, and Δ*R*/(∂*R*/∂*N*_eff_) versus *N*_eff_ under a 20 V square-wave voltage at 6 Hz for TM mode. (e–h) For **P5F-AJLZ53**-35%, the ATR measurement and calculation procedures were performed following the same analytical sequence (a–d) as those used for **P4F1-AJLZ53**-35%.

The ATR measurements demonstrated an effective EO activity (*n*_TM_^3^ *r*_33_) of 1169 pm/V at 1306 nm and 576 pm/V at 1541 nm for **P4F1-AJLZ53**-35%, representing one of the highest EO performances for guest–host polymers measured by ATR with explicit optical birefringence using the standard Metricon 2010/M prism coupler. The larger tensor ratio observed at 1306 nm for **P4F1-AJLZ53**-35% compared to that at 1541 nm may arise from the contribution of the imaginary component of the EO response at these two wavelengths. This finding underscores the importance of precise ATR characterization of tensor components at telecom wavelengths, which cannot be obtained accurately using the simple reflection technique.

The modulated ATR spectra of poled **P4F1-AJLZ53**-35% exhibited distinct mode splitting in TE polarization at the frequencies of 120 and 600 Hz (Figure 6a), and highly asymmetrical modulated optical signals in TM polarization (Figure 6d), reflecting exceptional EO properties of the material. The calculated *r*-coefficients from high-frequency measurements are consistent with the low-frequency analysis (Table S3). The unchanged ATR spectra for poled **P4F1-AJLZ53**-35% from 120 Hz to 20 kHz confirm a stable electronic polarization mechanism of modulation at high frequencies (Figure S5).

The alignment stability of the poled films was evaluated through a thermal annealing test conducted at 85 °C, during which changes in optical birefringence and the corresponding noncentrosymmetric order parameter were monitored. After thermal annealing at 85 °C for 500 h, the poled film of **P4F1-AJLZ53-**35% retained 82% of the initial polar order, and its stability is significantly better than the result of 69% for the poled **PC-AJLZ53-**35% (Figure 7b).^41,42^ The stability improvement is mainly contributed by higher *T*_g_ (or the highest poling temperatures) of the formulated guest–host polymers, and nonclassical H-bonds from fluorophenyl side chains in **P4F1** copolymer. These remarkable results are a great demonstration of the advantage offered by nonclassical H-bonds from fluorophenyl side chains that balance intermolecular interactions, elastic modulus, hardness, orientational polarization, and thermal stability, thereby optimizing the key properties of guest–host EO polymers for photonic applications.

**Figure 7:**
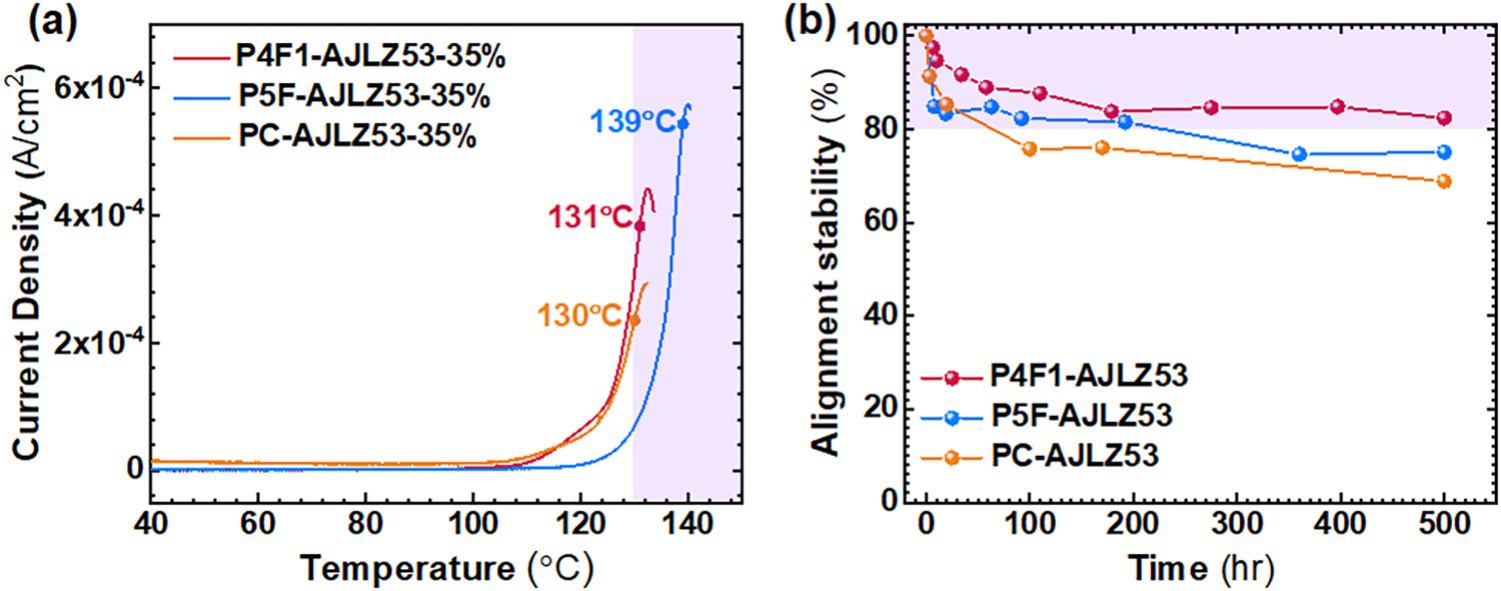
(a) Leak through currents (LTC) profiles for poled guest–host polymer films at the heating rate of 10 C/min under the field strength of ∼90 V/μm; (b) Thermal stability testing of poled **P4F1-AJLZ53**, **P5F-AJLZ53**, and **PC-AJLZ53** films with 35 wt % of chromophore loading at 85 °C for 500 h.

## Conclusions

This study provides a comprehensive demonstration of how singular fluorophenyl synthons, through weak H-bonds, can facilitate the development of highly efficient and thermally stable supramolecular guest–host EO polymers. It effectively addresses three critical yet often conflicting requirements: high rotational freedom of dipolar PPT-phores during poling, excellent mechanical properties against stress cracking, and temporal stability of aligned PPT-phores against thermal relaxation during operation. We have demonstrated that weak C–H···X (X = O, F) interactions significantly enhance the orientational order, mechanical properties, and thermal stability of guest–host EO polymers. Remarkably, **P4F1-AJLZ53** and **P5F1-AJLZ53** exhibited some of the highest *r*_33_ and *n* ^3^ *r*_33_ values at telecom wavelengths of 1306 and 1541 nm, as verified by ATR measurements, while maintaining a stable EO response at the elevated temperature of 85 °C. These properties are comparable to or better than those of polycarbonate-based benchmark EO polymers.

The study exemplifies the effectiveness of a bottom-up construction strategy based on weak hydrogen-bonding interactions. Although there is a lack of direct characterization techniques capable of probing weak intermolecular interactions within amorphous polymers, we overcame this challenge by precisely controlling the position and number of fluorine atoms of supramolecular synthons to fine-tune the weak interactions of functional EO polymers. The consistent structure–property relationship, verified by comprehensive characterizations, confirms that such weak interactions remain effective in PPT-phore-doped polymer matrices and enable the development of high-performance, thermally stable guest–host EO polymers.

Beyond their exceptional EO properties and straightforward synthesis, our systematic study of the structure–property relationship within this series of new guest–host EO polymers underscores the critical advantages of weak H-bond interactions facilitated by two less dipolar fluorophenyl synthons. These interactions are dispersive, flexible, and entropically stabilized, offering significant benefits over classical rigid π-stacking interactions in the design of high-performance supramolecular EO polymers.

Looking ahead, we anticipate that further exploration and investigation will strengthen the research in OEO materials as a unique and rapidly advancing platform in supramolecular chemistry and molecular photonics. The roles of TCF-based push–pull chromophores, which engage both strong electrostatic interactions and weak H-bonds,^42–47^ merit particular attention in future research. We hope that nonclassical hydrogen bonding interactions hold great potential as a versatile tool of supramolecular chemistry in the rational design of highly efficient, thermally stable supramolecular EO polymers for photonic applications.

## Supplementary Material







## Notes

Experimental Section, synthetic details and characterizations, NMR and HRMS spectra, DFT calculations, and modulated ATR spectra (PDF)
Single-crystal X-ray diffraction data of compound **M4F1** (CIF)
Single-crystal X-ray diffraction data of compound **M4F2** (CIF)
