# Supplementary material for: Supramolecular Maleimide–Styrene Copolymers with Fluorophenyl Side Chains for High-Performance Guest–Host Electro-Optic Materials
Source: Precis Chem. 2026 Feb 17;4(7):973–84. doi: 10.1021/prechem.5c00365 (PMC13417514; doi:10.1021/prechem.5c00365)
Supplement: Supplementary file 1 [file pc5c00365_si_001.pdf]

Supporting Information

Supramolecular Maleimide–Styrene Copolymers with Fluorophenyl Side Chains for High-Performance Guest-Host Electro-Optic Materials

*Danning Lyu,[1,2] Shivani Pathania,[1] and Jingdong Luo[1,2]**

[1] Department of Chemistry, City University of Hong Kong, Hong Kong SAR, China

[2] Shenzhen Research Institute, City University of Hong Kong, Shenzhen 518057, China

* Email: jingdluo@cityu.edu.hk

Contents

Materials and methods S2
Poling and ATR measurements for guest-host organic thin films S3
Synthesis of the monomers and polymers S4
DFT calculations for the four maleimido monomers by Guassian 16 .... S10
The macroscopic images of the fabricated thin films and UV-*vis*-NIR spectra for each system S11
ATR spectra from Metricon 2010/M and data processing S13
NMR and HRMS spectra of the maleimide intermediates, monomers, and polymers S16
X-ray data of the two maleimido monomers S31
Molecular weight characterization of polymers S32
EO analyses under high-frequency and low-frequency modulation voltages S33
Summary of methods for calculating EO coefficients from ATR technique S34
Calculation of order parameters of poled films from the poling-induced optical birefringences S36

Materials and methods

All chemicals were purchased from Energy Chemical or Sigma-Aldrich and used as received unless otherwise mentioned. ^{1}H NMR and ^{13}C NMR spectra were recorded on a Bruker 300 MHz "AVANCE III HD" Nuclear Magnetic Resonance System (NMR-300), Bruker 400 MHz "AVANCE III" Nuclear Magnetic Resonance System (NMR-400), and Bruker 600 MHz "AVANCE III HD" Nuclear Magnetic Resonance System (NMR-600). High-resolution mass spectrometry (HRMS) data were acquired using a Thermo Scientific LTQ Orbitrap XL mass spectrometer. The molecular weight and other properties of the polymers were determined using a Waters E2695 gel permeation chromatography (GPC) system operating at room temperature. For thin film preparation, both polymers and chromophores were dissolved in 1,1,2-trichloroethane (TCE). The films were spin-coated onto patterned indium tin oxide (ITO) glass substrates using a SPIN-PROCESS CONTROLLER instrument. Differential scanning calorimetry (DSC) measurements were performed on a PerkinElmer STA 6000 Simultaneous Thermal Analyzer with a heating rate of 10 °C/min. The UV-*vis*-NIR spectra were recorded with Ultra-Violet-Visible Scanning Spectrophotometer (Shimadzu 1700) and Ultra-Violet-Visible-Near Infrared Spectrophotometer with Integrating Sphere (PE Lamda 1050). Surface morphology was characterized using scanning electron microscopy (SEM, Thermo Scientific Apreo 2). Nanoindentation tests were performed on a KLA Instruments iMicro nanoindenter equipped with a Berkovich tip by the Testing Technology Center of Materials and Devices, Tsinghua Shenzhen International Graduate School, China. DFT calculations using the Gaussian 16 package were carried out at the level of B3LYP/6-31+G(d,p) for ground-state geometry optimization.

Poling and ATR measurements for guest-host organic thin films

For studying the EO property derived from the chromophores, the guest-host organic EO polymer films were prepared by mixing a benchmark chromophore, Alex Jen-Luo-Zhou-53 (AJLZ53), with the host polymer polycarbonate (PC), P4F1, and P5F at a high loading density of 35 wt% in trichloroethylene (TCE) solution. The resulting solutions were filtered through a 0.2 μm PTFE filter and spin-coated onto indium tin oxide (ITO) glass substrates. After the soft baking, films of doped polymers were baked in a vacuum oven overnight at 75 °C to ensure the complete removal of residual solvent. Thicknesses of films were measured by DektakXT Stylus Profiler and further confirmed on the subsequent optical measurement by a commercial prism-coupler system (Metricon 2010/M). Then, using the Desk V HP Sputter Unit (Denton Vacuum LLC), a thin layer (~10 nm) of semi-transparent gold was sputtered onto the films as a top electrode for contact poling and subsequent EO measurements. The electric field poling of films was conducted at a central processor-controlled Mettler FP82 hot stage. The poled thin films were prepared by heating the samples from 40 °C to the T_g of EO polymers at a constant rate of 10 °C min^{-1} under a given field strength, supplied and monitored by Keithley 2657A.

After the poling, the refractive indices and reflectivity spectra of the poled films were determined using the attenuated total reflection (ATR) method in a slab waveguide geometry by Metricon 2010/M at the wavelengths of 1306 nm and 1541 nm. The modulated reflectivity spectra of poled films were recorded by applying modulation voltages to the poled films through a low-distortion function generator DS360 from Stanford Research Systems. The ATR spectra of poled films were exported and analyzed/plotted by Origin for the calculation of the r_{13} and r_{33} values.

Synthesis of the monomers and polymers

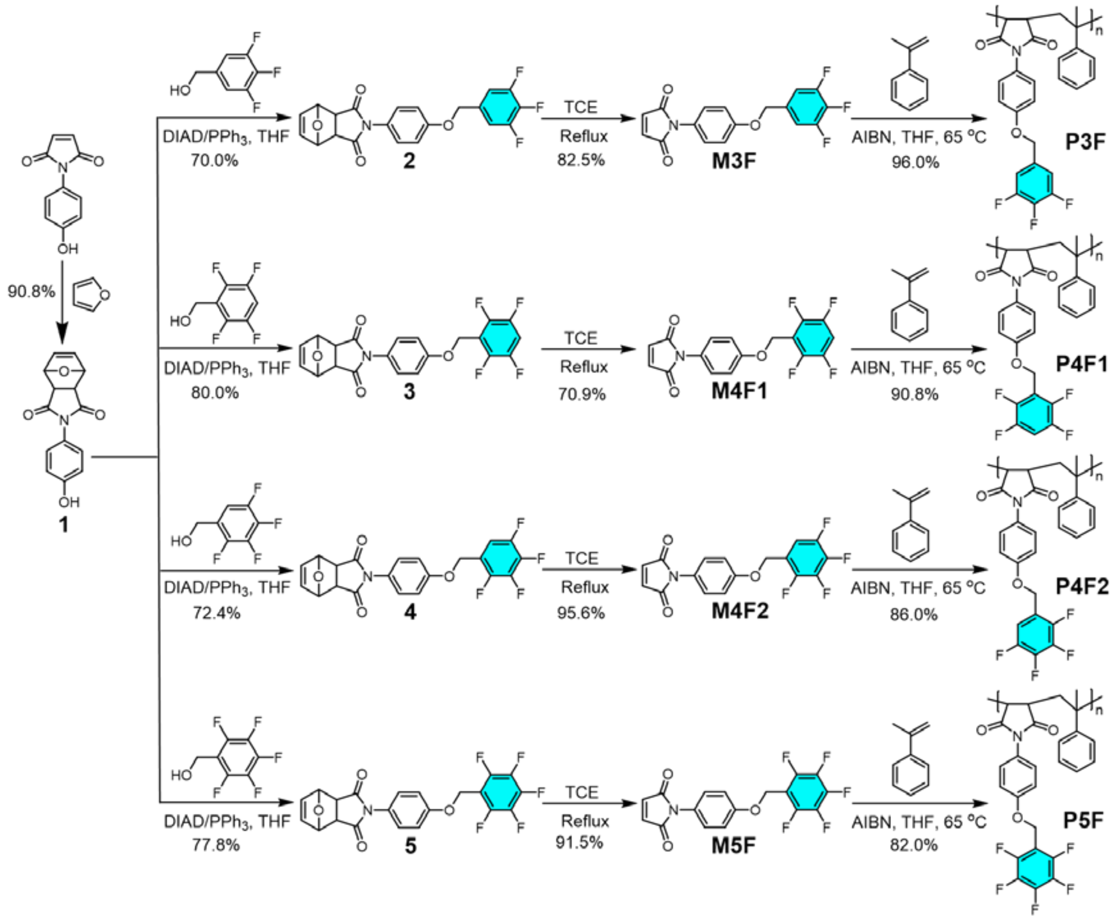

(1) Synthesis of compound **1**

1-(4-Hydroxyphenyl)-1H-pyrrole-2,5-dione (1 g, 5.29 mmol) was dissolved in 5 mL acetonitrile (ACN). Under a nitrogen atmosphere, furan (0.57 mL, 7.93 mmol) was added, and the reaction was carried out at 90 °C for 12 hours. After the reaction, it was purified by column chromatography with the eluent of hexane: ethyl acetate (EA, 2:1) to afford the product. (1.23 g, yield: 90.8%) ^{1}H NMR (300 MHz, DMSO-d_6) δ 9.78 (s, 1H), 7.00 – 6.94 (m, 2H), 6.86 – 6.80 (m, 2H), 6.60 (s, 2H), 5.23 (t, J = 1.0 Hz, 2H), 3.04 (s, 2H). ^{13}C NMR (75 MHz, DMSO-d_6) δ 176.51, 157.79, 137.06, 128.53, 123.69, 115.90, 81.17, 47.72.

(2) Synthesis of compound **2**

Compound 1 (514 mg, 2 mmol), 3,4,5-trifluorobenzaldehyde alcohol (486 mg, 3 mmol), and triphenylphosphine (PPh_3, 787 mg, 3 mmol) were dissolved in 5 mL of dry tetrahydrofuran (THF). The flask was placed in the ice bath, and diisopropyl

azodicarboxylate (DIAD, 0.6 mL, 3 mmol) was added to the flask through the injection syringe. It reacted for 12 hours. After the reaction, it was purified by column chromatography with the eluent of hexane: ethyl acetate (EA, 4:1) to afford the product. (560 mg, yield: 70%) ^{1}H NMR (400 MHz, Chloroform-*d*) δ 7.28 – 7.19 (m, 2H), 7.12 – 6.97 (m, 4H), 6.59 (s, 2H), 5.41 (s, 2H), 5.02 (s, 2H), 3.03 (s, 2H).

(3) Synthesis of compound **3**

Compound 1 (514 mg, 2 mmol), 2,3,5,6-tetrafluorobenzyl alcohol (540.3 mg, 3 mmol), and triphenylphosphine (PPh_3, 787 mg, 3 mmol) were dissolved in 5 mL of dry THF. The flask was placed in the ice bath, and diisopropyl azodicarboxylate (DIAD, 0.6 mL, 3 mmol) was added to the flask through the injection syringe. It reacted for 12 hours. After the reaction, it was purified by column chromatography with the eluent of hexane: ethyl acetate (EA, 8:1) to afford the product. (670 mg, yield: 80%) ^{1}H NMR (300 MHz, Chloroform-*d*) δ 7.28 – 7.22 (m, 2H), 7.22 – 7.13 (m, 1H), 7.11 – 7.06 (m, 2H), 6.59 (t, J = 1.0 Hz, 2H), 5.42 (t, J = 1.0 Hz, 2H), 5.19 (t, J = 1.5 Hz, 2H), 3.03 (s, 2H).

(4) Synthesis of compound **4**

Compound 1 (514 mg, 2 mmol), 2,3,4,5-tetrafluorobenzyl alcohol (540.3 mg, 3 mmol), and triphenylphosphine (PPh_3, 787 mg, 3 mmol) were dissolved in 5 mL of dry THF. The flask was placed in the ice bath, and diisopropyl azodicarboxylate (DIAD, 0.6 mL, 3 mmol) was added to the flask through the injection syringe. It reacted for 12h. After the reaction, it was purified by column chromatography with the eluent of hexane: ethyl acetate (EA, 8:1) to afford the product. (607 mg, yield: 72.4%) ^{1}H NMR (300 MHz, Chloroform-*d*) δ 7.29 – 7.23 (m, 2H), 7.17 (ddd, J = 10.1, 5.6, 2.3 Hz, 1H), 7.11 – 7.02 (m, 2H), 6.59 (t, J = 0.9 Hz, 2H), 5.41 (t, J = 0.9 Hz, 2H), 5.12 (s, 2H), 3.03 (s, 2H).

(5) Synthesis of compound **5**

Compound 1 (514 mg, 2 mmol), 2,3,4,5,6-pentafluorobenzyl alcohol (594.3 mg, 3 mmol), and triphenylphosphine (PPh_3, 787 mg, 3 mmol) were dissolved in 5 mL of dry THF. The flask was placed in the ice bath, and diisopropyl azodicarboxylate (DIAD, 0.6 mL, 3 mmol) was added to the flask through the injection syringe. It reacted for 12

hours. After the reaction, it was purified by column chromatography with the eluent of hexane: ethyl acetate (EA, 8:1) to afford the product. (652 mg, yield: 77.8%) ^{1}H NMR (300 MHz, Chloroform-*d*) δ 7.29 – 7.21 (m, 2H), 7.13 – 6.99 (m, 2H), 6.59 (t, *J* = 1.0 Hz, 2H), 5.42 (t, *J* = 1.0 Hz, 2H), 5.16 (t, *J* = 1.6 Hz, 2H), 3.03 (s, 2H).

(6) Synthesis of compound **M3F**

Compound 2 (507 mg) was dissolved in 10 mL of trichloroethane and refluxed at 116 °C for 20 hours under a nitrogen atmosphere. After the reaction, it was purified by column chromatography with the eluent of hexane: ethyl acetate (EA, 4:1) to afford the product. (347.1 mg, yield: 82.5%) ^{1}H NMR (300 MHz, Chloroform-*d*) δ 7.29 (d, *J* = 2.3 Hz, 1H), 7.27 (d, *J* = 2.2 Hz, 1H), 7.13 – 7.01 (m, 4H), 6.87 (s, 2H), 5.03 (s, 2H). ^{13}C NMR (151 MHz, Chloroform-*d*) δ 169.70, 157.55, 151.39, 139.33, 134.20, 133.01, 127.69, 124.67, 115.37, 111.14, 68.51. ^{19}F NMR (565 MHz, Chloroform-*d*) δ -133.55, -161.01. HRMS (ESI) *m*/*z* calcd for $C_{17}H_{10}F_3NO_3H^+$ $(M+H)^+$ 334.06128, found 334.06912.

(7) Synthesis of compound **M4F1**

Compound 3 (660 mg) was dissolved in 10 mL of trichloroethane and refluxed at 116 °C for 20 hours under a nitrogen atmosphere. After the reaction, it was purified by column chromatography with the eluent of hexane: ethyl acetate (EA, 4:1) to afford the product. (347.1 mg, yield: 70.9%) ^{1}H NMR (300 MHz, Chloroform-*d*) δ 7.30 (d, *J* = 6.7 Hz, 2H), 7.21 – 7.07 (m, 3H), 6.87 (s, 2H), 5.20 (t, *J* = 1.5 Hz, 2H). ^{13}C NMR (151 MHz, Chloroform-*d*) δ 169.70, 157.58, 145.86, 145.28, 134.21, 127.70, 124.82, 115.52, 115.42, 106.88, 58.07. ^{19}F NMR (565 MHz, Chloroform-*d*) δ -138.53 (dd, *J* = 22.1, 13.4 Hz), -142.87 (dd, *J* = 21.7, 13.4 Hz). HRMS (ESI) *m*/*z* calcd for $C_{17}H_9F_4NO_3H^+$ $(M+H)^+$ 352.05186, found 352.05844.

(8) Synthesis of compound **M4F2**

Compound 4 (590 mg) was dissolved in 10 mL of trichloroethane and refluxed at 116 °C for 20 hours under a nitrogen atmosphere. After the reaction, it was purified by column chromatography with the eluent of hexane: ethyl acetate (EA, 4:1) to afford the product. (472.5 mg, yield: 95.6%) ^{1}H NMR (300 MHz, Chloroform-*d*) δ 7.33 – 7.28 (m, 2H), 7.24 – 7.14 (m, 1H), 7.09 – 7.03 (m, 2H), 6.87 (s, 2H), 5.13 (s, 2H). ^{13}C NMR

(151 MHz, Chloroform-*d*) δ 169.67, 157.31, 147.32, 145.09, 140.66, 140.24, 134.21, 127.73, 124.88, 120.40, 115.32, 110.40, 62.64. ^{19}F NMR (565 MHz, Chloroform-*d*) δ -138.47 (dd, *J* = 20.9, 12.7 Hz), -143.76 (ddd, *J* = 20.9, 12.8, 3.3 Hz), -155.34 (t, *J* = 20.0 Hz), -155.69 (td, *J* = 20.4, 3.2 Hz). HRMS (ESI) *m*/*z* calcd for $C_{17}H_9F_4NO_3H^+$ $(M+H)^+$ 352.05186, found 352.05936.

(9) Synthesis of compound **M5F**

Compound 4 (560 mg) was dissolved in 10 mL of trichloroethane and refluxed at 116 °C for 20 hours under a nitrogen atmosphere. After the reaction, it was purified by column chromatography with the eluent of hexane: ethyl acetate (EA, 4:1) to afford the product. (432.2 mg, yield: 91.5%) ^{1}H NMR (600 MHz, Chloroform-*d*) δ 7.31 – 7.28 (m, 2H), 7.10 – 7.04 (m, 2H), 6.87 (s, 2H), 5.17 (t, *J* = 1.6 Hz, 2H). ^{13}C NMR (151 MHz, Chloroform-*d*) δ 169.66, 157.42, 145.75, 141.86, 137.59, 134.20, 127.70, 124.97, 115.42, 109.86, 57.68. ^{19}F NMR (565 MHz, Chloroform-*d*) δ -142.14 – -142.24 (m), -152.40 (t, *J* = 20.7 Hz), -161.33 – -161.47 (m).

(10) Synthesis of compound **P3F**

In a Pyrex glass tube, 363 mg (1.1 mmol) of M3F and 0.14 mL (1.1 mmol) of *α*-methylstyrene, and 5.4 mg (0.033 mmol) of AIBN as radical initiator were dissolved in 1 mL of THF. The tube was cooled to liquid N_2 temperature, evacuated, filled with N_2, and thawed. The above procedure was repeated three times; then, the tube was cooled to liquid N_2 temperature and sealed under vacuum. Copolymerization was performed at 65 °C for 24 hours. After the copolymerization, the content of the tube was poured into a large amount of methanol to precipitate the copolymer. The copolymer thus isolated was purified by pouring its DCM solution into methanol. The purified copolymer was dried in vacuo at 75 °C for 48 hours. Yield: 473 mg (96%). ^{1}H NMR (400 MHz, DMSO-*d*$_6$) δ 7.25 (br d, *J* = 130.6 Hz, 11H), 5.12 (br d, *J* = 15.7 Hz, 2H), 2.68 (br s, 1H), 2.44 – 0.22 (br m, 6H). ^{19}F NMR (565 MHz, DMSO-*d*$_6$) δ -134.90 (d, *J* = 21.5 Hz), -162.52.

(11) Synthesis of compound **P4F1**

In a Pyrex glass tube, 385 mg (1.1 mmol) of M4F1 and 0.14 mL (1.1 mmol) of *α*-methylstyrene, and 5.4 mg (0.033 mmol) of AIBN as radical initiator were dissolved in

1 mL of THF. The tube was cooled to liquid N_2 temperature, evacuated, filled with N_2, and thawed. The above procedure was repeated three times; then, the tube was cooled to liquid N_2 temperature and sealed under vacuum. Copolymerization was performed at 65 ^oC for 24 hours. After the copolymerization, the content of the tube was poured into a large amount of methanol to precipitate the copolymer. The copolymer thus isolated was purified by pouring its DCM solution into methanol. The purified copolymer was dried in vacuo at 75 ^oC for 48 hours. Yield: 468 mg (90.8%). ^1H NMR (400 MHz, DMSO-d_6) δ 7.94 (br d, J = 9.3 Hz, 1H), 6.93 (br, d, J = 89.3 Hz, 9H), 5.20 (br s, 2H), 2.21 – 0.30 (br, m, 7H). ^{19}F NMR (565 MHz, DMSO-d_6) δ -139.09, -143.39.

(12) Synthesis of compound **P4F2**

In a Pyrex glass tube, 385 mg (1.1 mmol) of M4F2 and 0.14 mL (1.08 mmol) of *α*-methylstyrene, and 5.4 mg (0.033 mmol) of AIBN as radical initiator were dissolved in 1 mL of THF. The tube was cooled to liquid N_2 temperature, evacuated, filled with N_2, and thawed. The above procedure was repeated three times; then, the tube was cooled to liquid N_2 temperature and sealed under vacuum. Copolymerization was performed at 65 ^oC for 24 hours. After the copolymerization, the content of the tube was poured into a large amount of methanol to precipitate the copolymer. The copolymer thus isolated was purified by pouring its DCM solution into methanol. The purified copolymer was dried in vacuo at 75 ^oC for 48 hours. Yield: 443 mg (86%). ^1H NMR (400 MHz, DMSO-d_6) δ 7.60 (br s, 1H), 6.93 (br, d, J = 122.8 Hz, 9H), 5.17 (br s, 2H), 2.24 – 0.62 (br, m, 7H). ^{19}F NMR (565 MHz, DMSO-d_6) δ -139.60, -143.06, -156.41 (d, J = 34.5 Hz).

(13) Synthesis of compound **P5F**

In a Pyrex glass tube, 400 mg (1.08 mmol) of M5F and 0.14 mL (1.08 mmol) of *α*-methylstyrene, and 5.3 mg (0.03 mmol) of AIBN as radical initiator were dissolved in 1 mL of THF. The tube was cooled to liquid N_2 temperature, evacuated, filled with N_2, and thawed. The above procedure was repeated three times; then, the tube was cooled to liquid N_2 temperature and sealed under vacuum. Copolymerization was performed at 65 ^oC for 24 hours. After the copolymerization, the content of the tube was poured into a large amount of methanol to precipitate the copolymer. The copolymer thus

isolated was purified by pouring its DCM solution into methanol. The purified copolymer was dried in vacuo at 75 ^{o}C for 48 hours. Yield: 434 mg (82%). ^{1}H NMR (400 MHz, chloroform-*d*) δ 6.99 (br, s, 9H), 5.11 (br s, 2H), 2.74 – 1.80 (br, m, 2H), 1.35 – 0.11 (br m, 5H). ^{19}F NMR (565 MHz, DMSO-d_6) δ -142.99, -143.13, -153.28, -162.27.

DFT calculations for the four maleimido monomers by Guassian 16

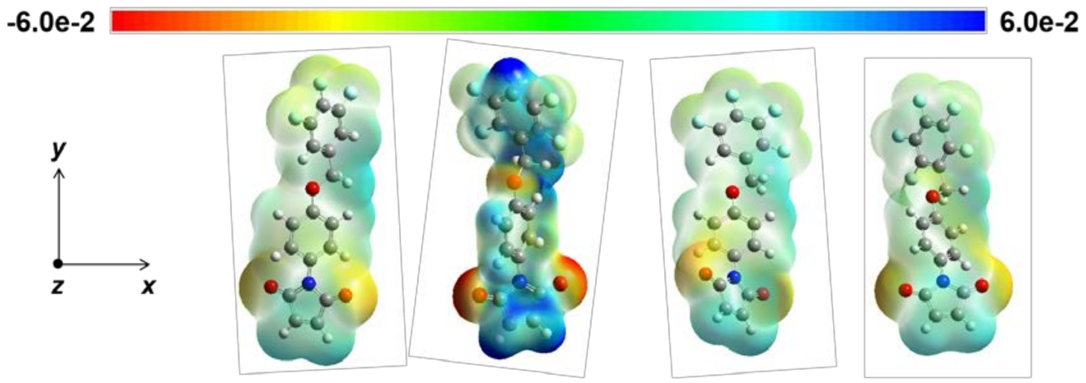

	$C_{17}H_{10}F_3NO_3$ (M3F)	$C_{17}H_9F_4NO_3$ (M4F1)	$C_{17}H_9F_4NO_3$ (M4F2)	$C_{17}H_8F_5NO_3$ (M5F)
μ (Debye)	3.4478	1.3580	3.4081	2.5198

Fig. S1 The electrostatic potential (ESP) surface, dipole moment (μ) of *N*-phenylmaleimido monomers with fluorobenzyl ether group derivatives (**M3F**, **M4F1**, **M4F2**, and **M5F**) by DFT.

The macroscopic images of the fabricated thin films and UV-*vis*-NIR spectra for each system

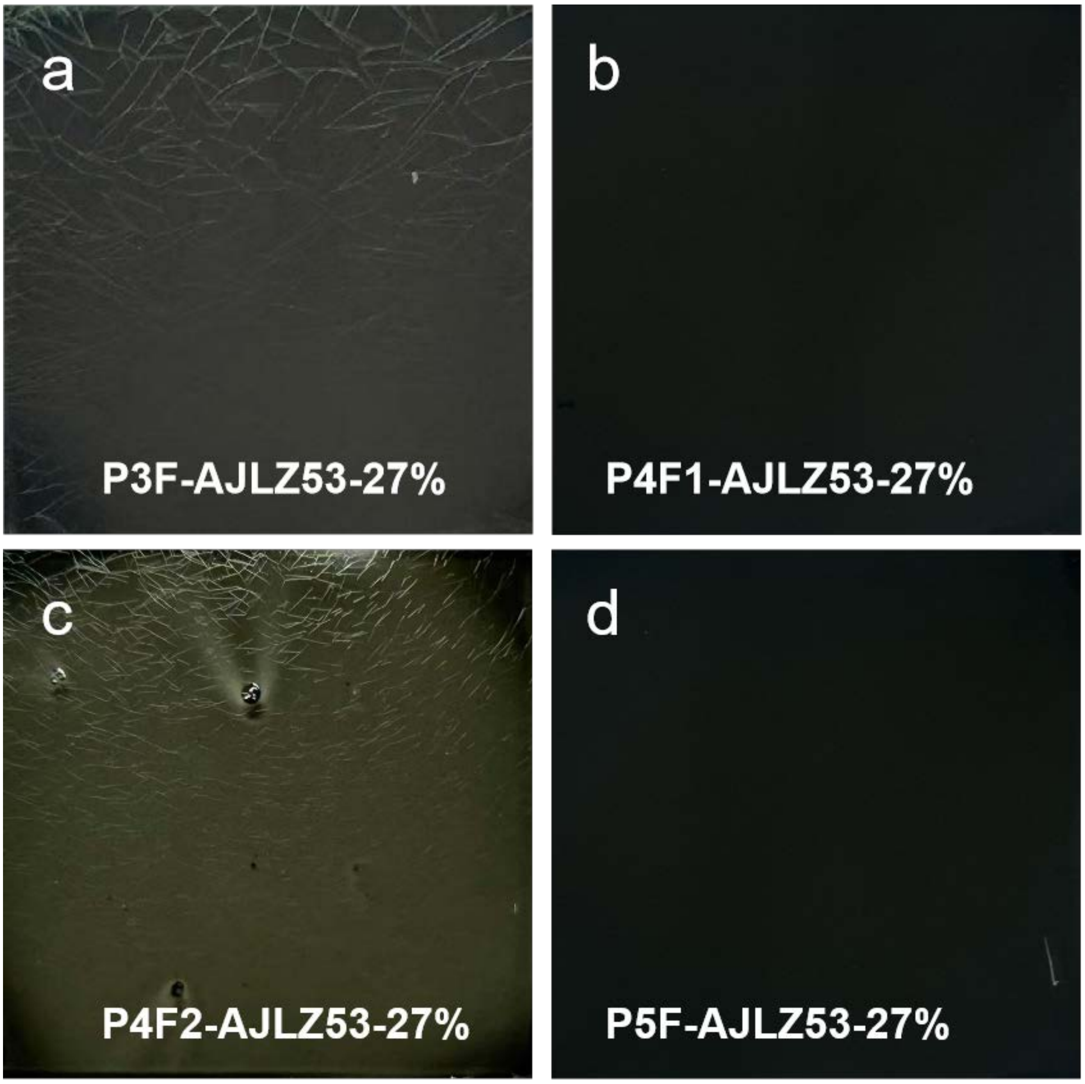

Fig. S2 The macroscopic images were acquired using an iPhone 14 smartphone camera under consistent LED lighting conditions. No post-processing was applied except for minor brightness adjustments to match the actual visual appearance. Under the same loading density, the four polymers exhibited distinct film-forming properties. Films prepared from **P3F** and **P4F2** showed pronounced cracking even when spun at a high speed of 1000 rpm, whereas **P4F1** and **P5F** formed thicker films that remained smooth and intact even at a lower speed of 600 rpm. (The actual dimensions of each film are 2.5 cm × 2.5 cm)

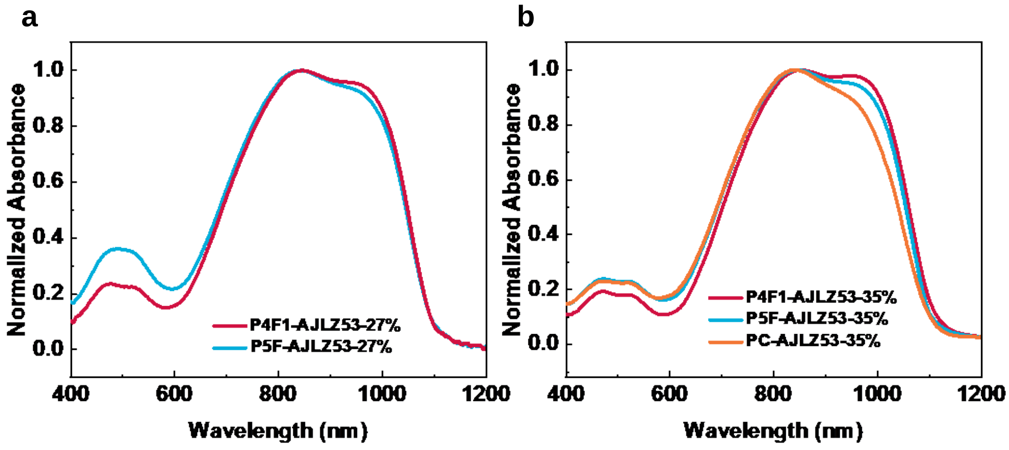

Fig. S3 UV-*vis*-NIR spectra of films with different loading densities.

ATR spectra from Metricon 2010/M and data processing

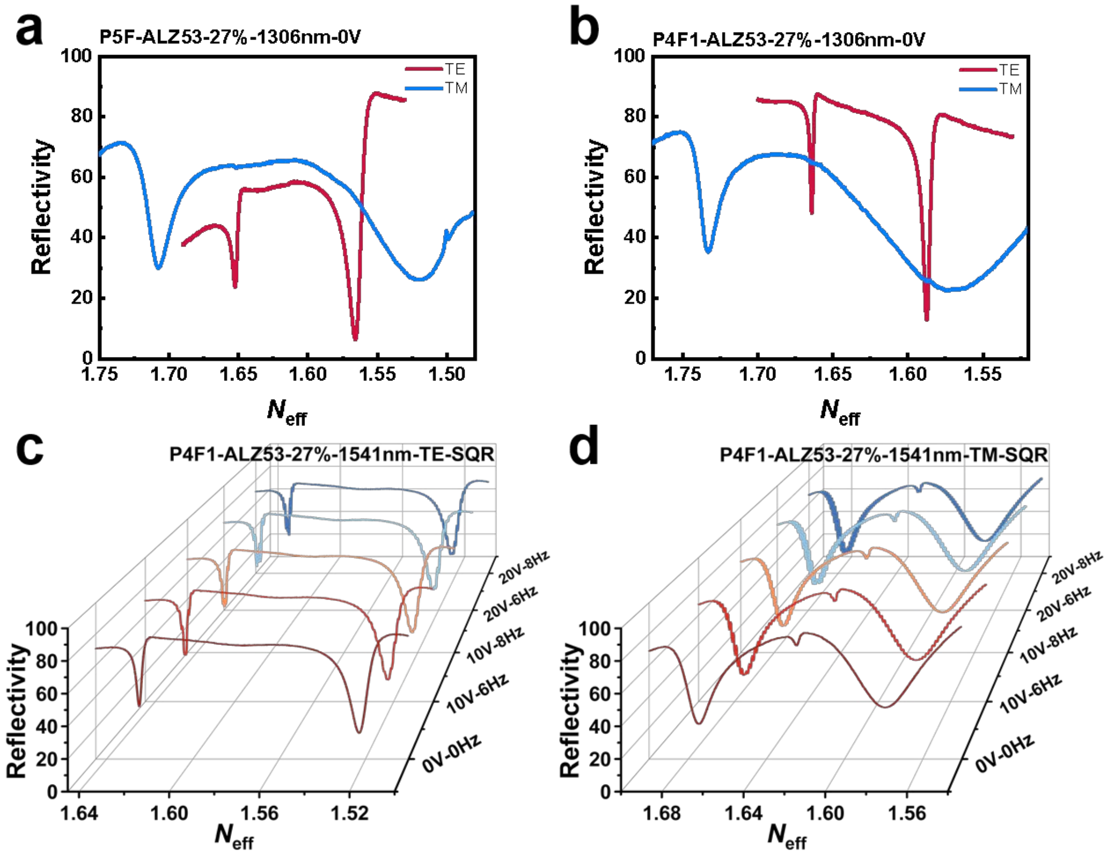

Fig. S4 Original ATR Spectra of **P5F-AJLZ53**-27%: (a) The change of reflectivity with the effective refractive index (N_{eff}) for the TE and TM modes at 0 V at 1306 nm. Original ATR Spectral Data of **P4F1-AJLZ53**-27%: (b) The change of reflectivity with the effective refractive index (N_{eff}) for the TE and TM modes at 0 V at 1306 nm. (c) In TE mode, the reflectivity as a function of the N_{eff}, unmodulated (0 V), and modulated by square-wave voltages of different frequencies and different voltages. (d) In TM mode, the reflectivity as a function of the N_{eff}, unmodulated (0 V) and modulated by square-wave voltages of different frequencies and different voltages.

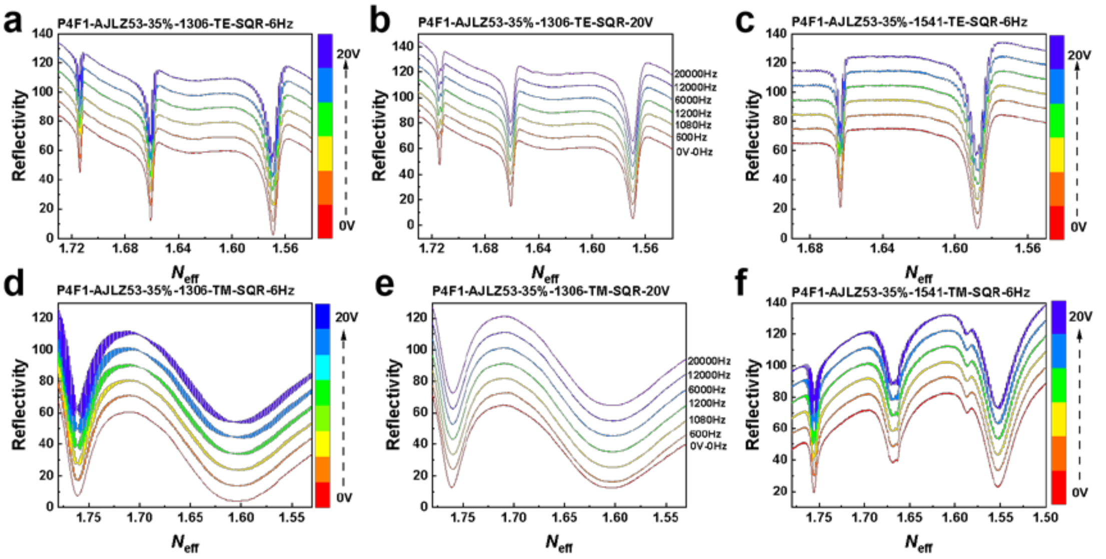

Fig. S5 Original ATR Spectra of **P4F1-AJLZ53**-35% at **1306 nm**: (a) The change of reflectivity with the effective refractive index (N_{eff}) for the TE modes when the square waves (0-20 V) power at 6 Hz were applied to the films. (b) The change of reflectivity with the effective refractive index (N_{eff}) for the TE modes when the square waves (0/20 V) power at different frequencies were applied to the films. (d) The change of reflectivity with the effective refractive index (N_{eff}) for the TM modes when the square waves (0-20 V) at 6 Hz were applied to the films. (e) The change of reflectivity with the effective refractive index (N_{eff}) for the TM modes when the square waves (0/20 V) power at different frequencies were applied to the films. Original ATR Spectral Data of **P4F1-AJLZ53**-35% at **1541 nm**: (c) The change of reflectivity with the effective refractive index (N_{eff}) for the TE modes when the square waves (0-20 V) power at 6 Hz were applied to the films. (f) The change of reflectivity with the effective refractive index (N_{eff}) for the TM modes when the square waves (0-20 V) at 6 Hz were applied to the films.

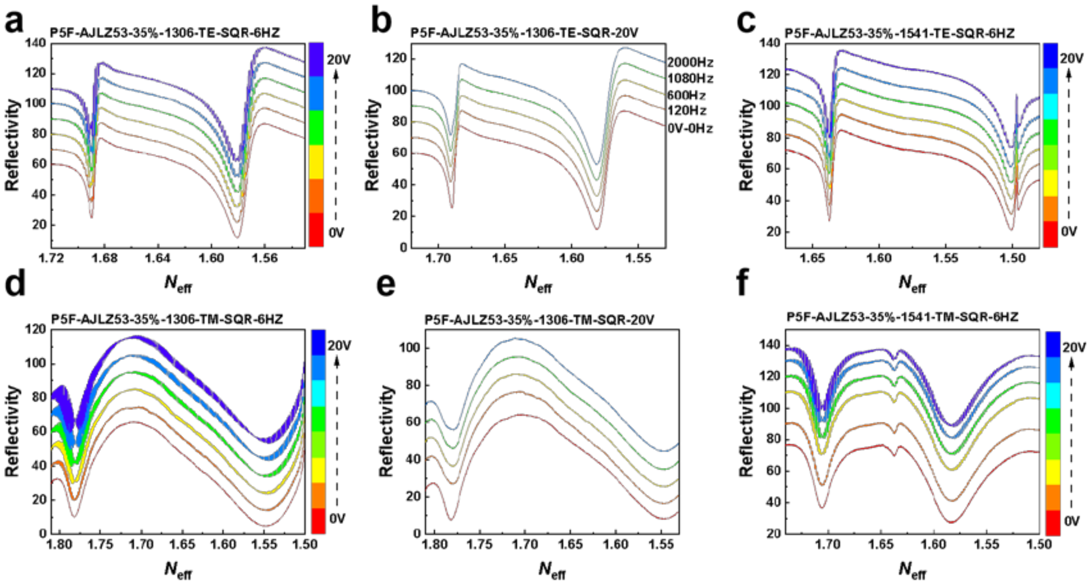

Fig. S6 Original ATR Spectra of **P5F-AJLZ53**-35% at **1306 nm**: (a) The change of reflectivity with the effective refractive index (N_{eff}) for the TE modes when the square waves (0-20 V) power at 6 Hz were applied to the films. (b) The change of reflectivity with the effective refractive index (N_{eff}) for the TE modes when the square waves (0/20 V) power at different frequencies were applied to the films. (d) The change of reflectivity with the effective refractive index (N_{eff}) for the TM modes when the square waves (0-20 V) at 6 Hz were applied to the films. (e) The change of reflectivity with the effective refractive index (N_{eff}) for the TM modes when the square waves (0/20 V) power at different frequencies were applied to the films. Original ATR Spectral Data of **P5F-AJLZ53**-35% at **1541 nm**: (c) The change of reflectivity with the effective refractive index (N_{eff}) for the TE modes when the square waves (0-20 V) power at 6 Hz were applied to the films. (f) The change of reflectivity with the effective refractive index (N_{eff}) for the TM modes when the square waves (0-20 V) at 6 Hz were applied to the films.

NMR and HRMS spectra of the maleimide intermediates, monomers, and polymers

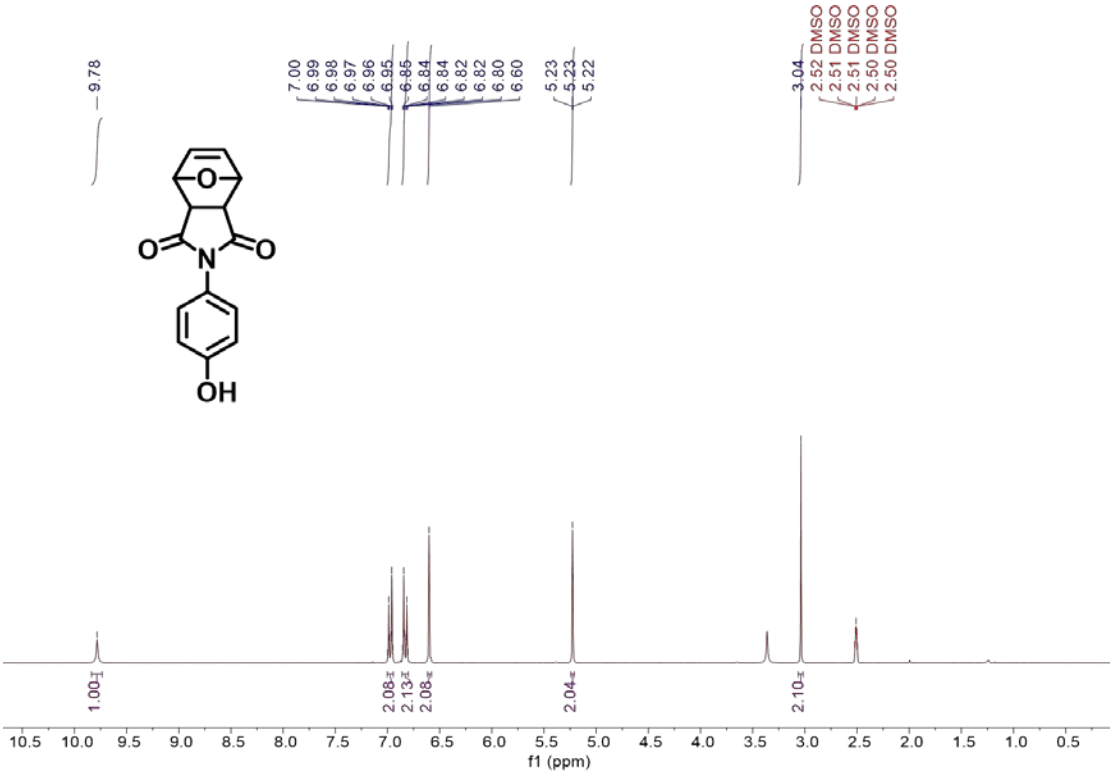

Fig. S7 ^{1}H NMR spectrum of compound 1 in DMSO.

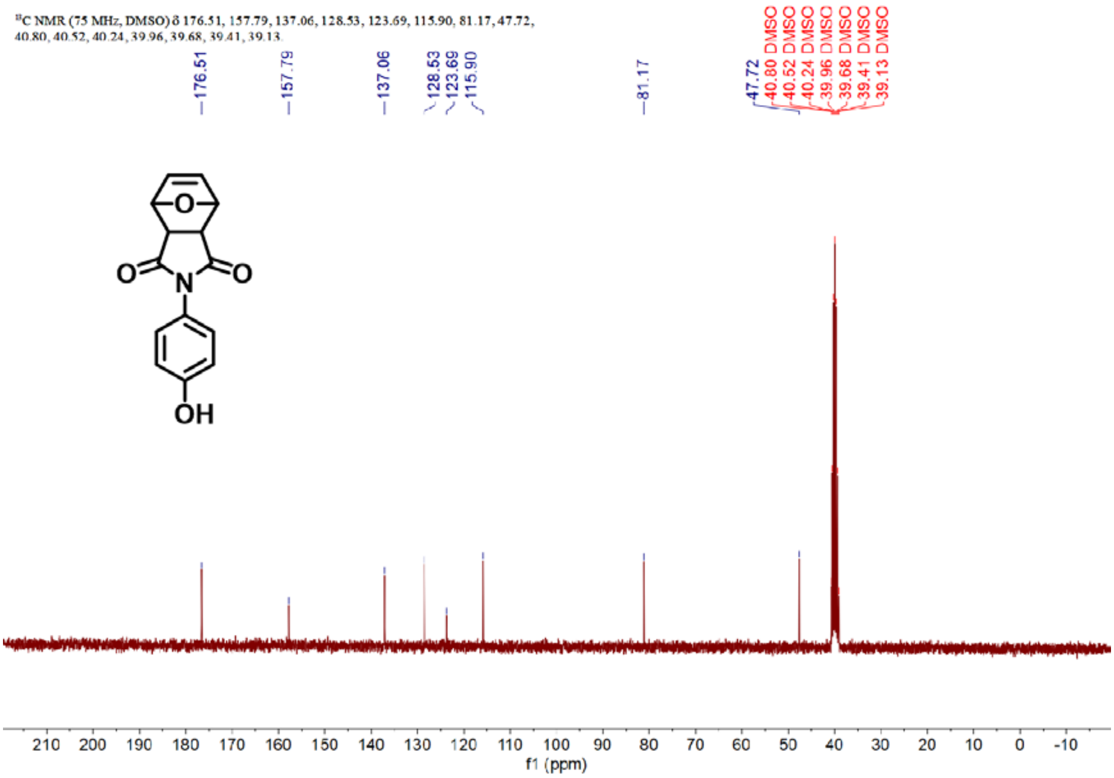

Fig. S8 ^{13}C NMR spectrum of compound 1 in DMSO.

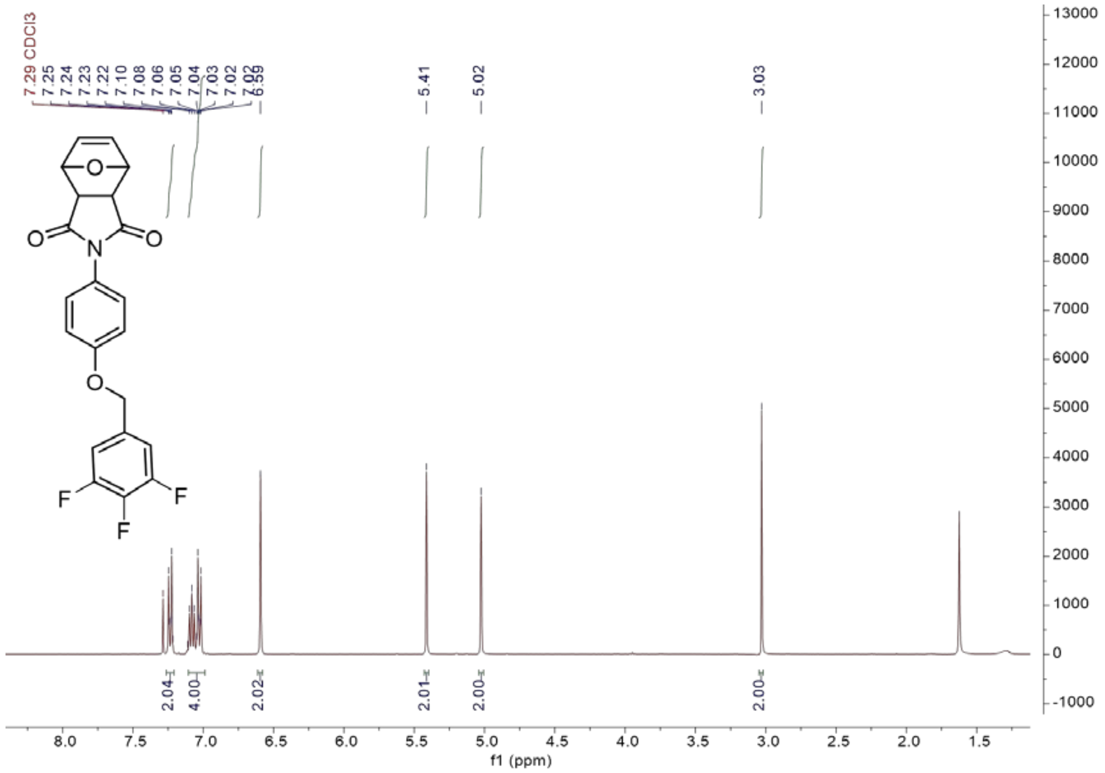

Fig. S9 ^{1}H NMR spectrum of compound 2 in $CDCl_3$.

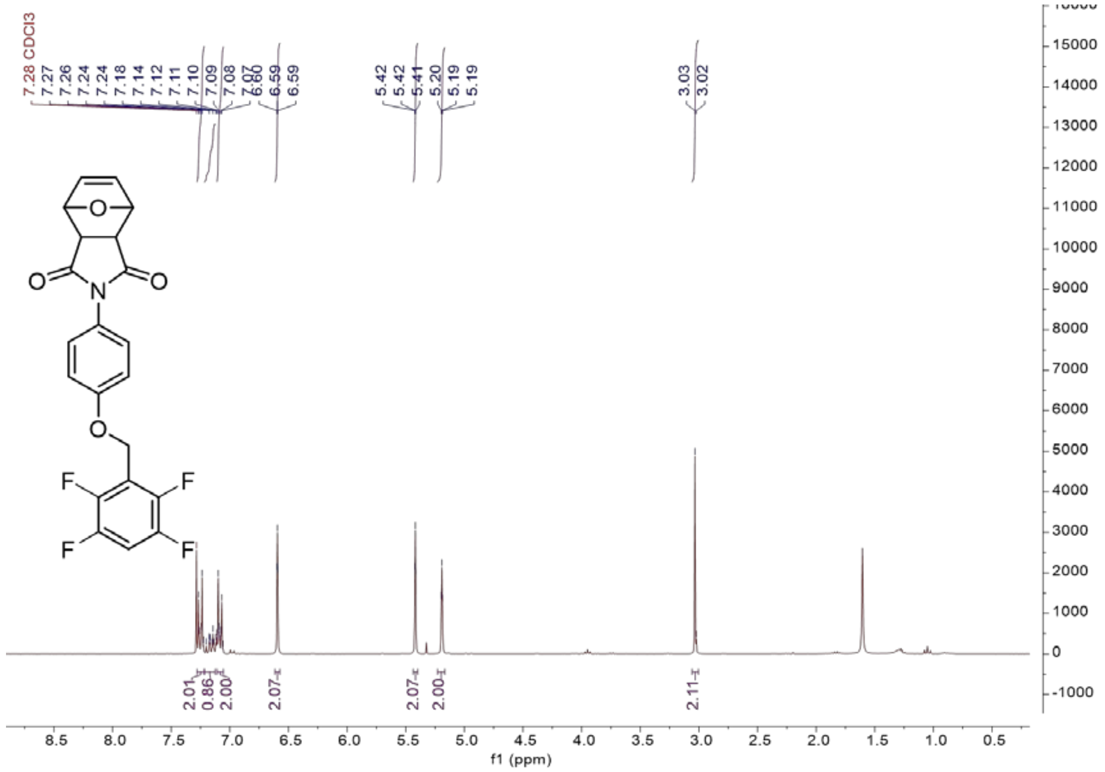

Fig. S10 ^{1}H NMR spectrum of compound 3 in $CDCl_3$.

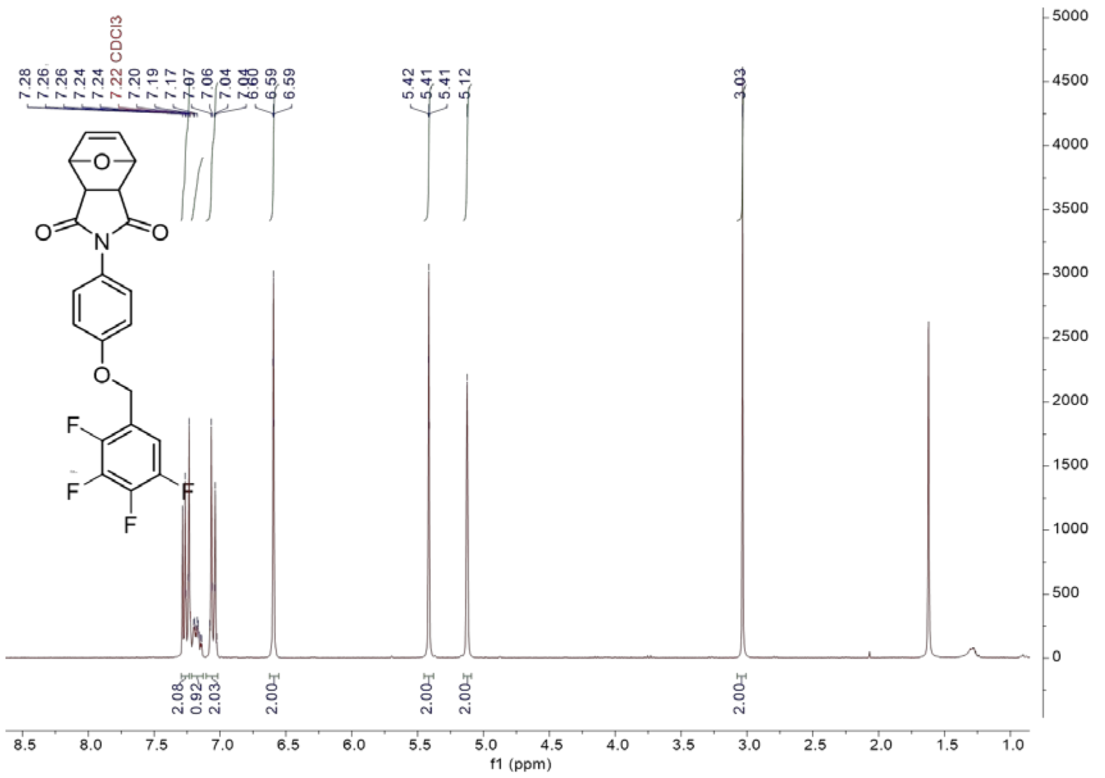

Fig. S11 ^{1}H NMR spectrum of compound 4 in $CDCl_3$.

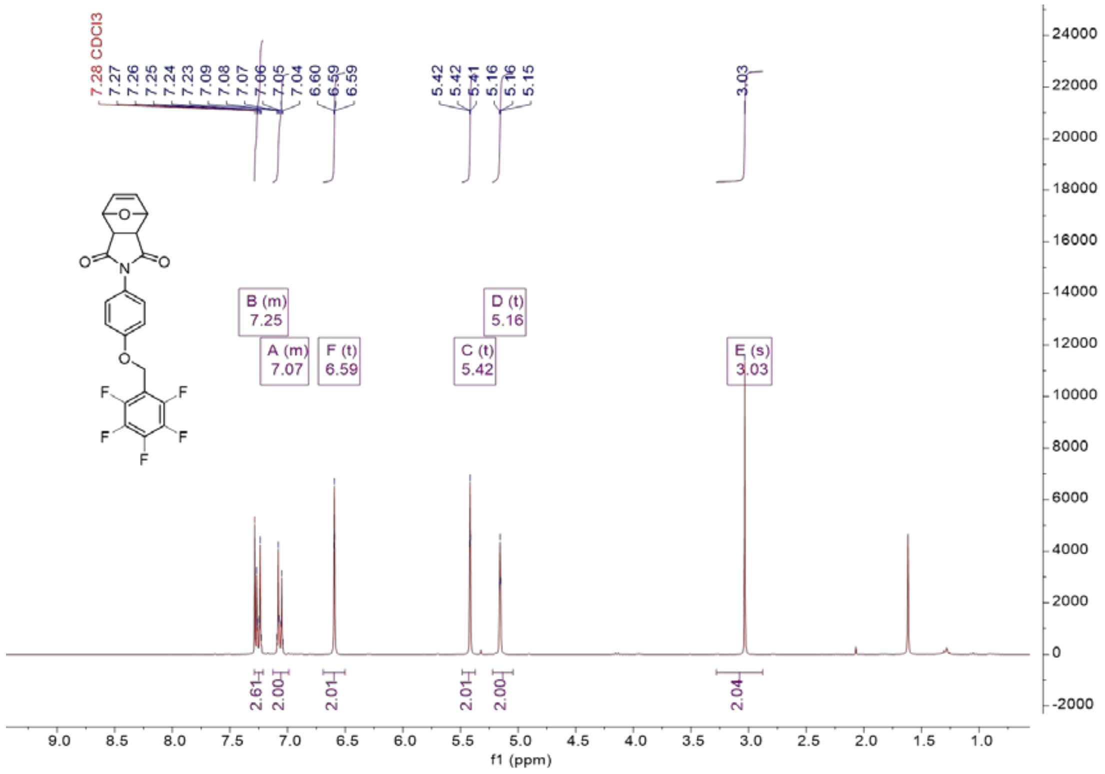

Fig. S12 ^{1}H NMR spectrum of compound 5 in $CDCl_3$.

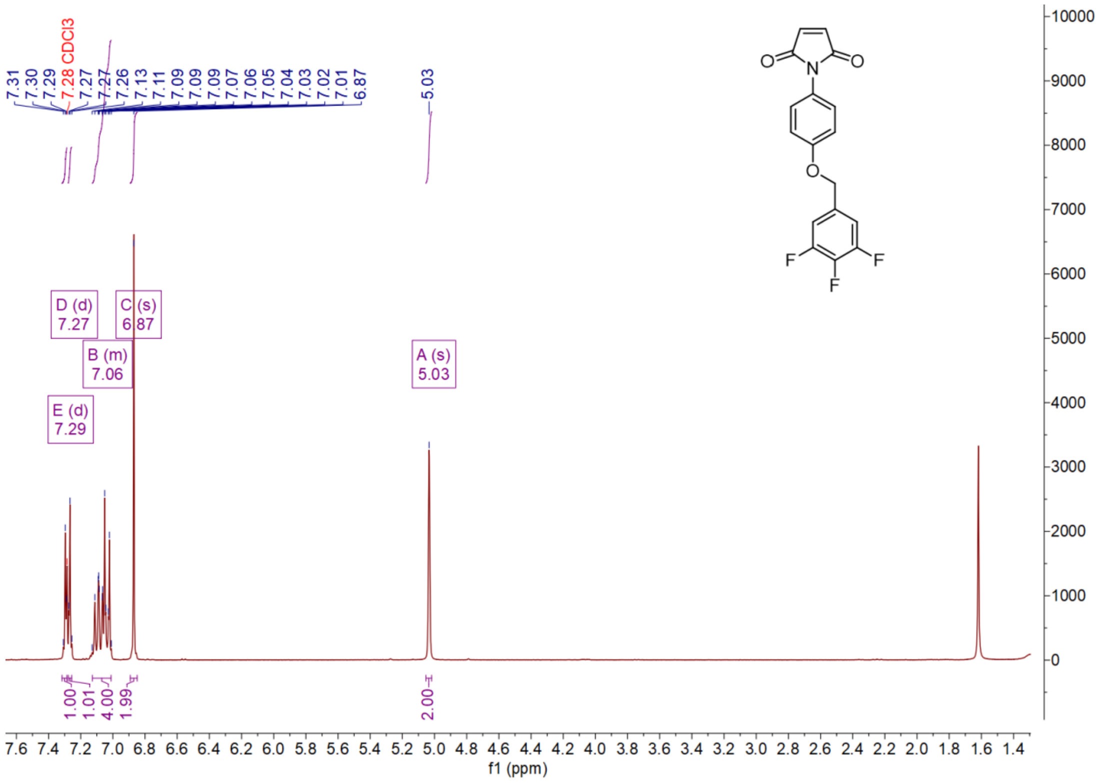

Fig. S13 ^{1}H NMR spectrum of M3F in $CDCl_3$.

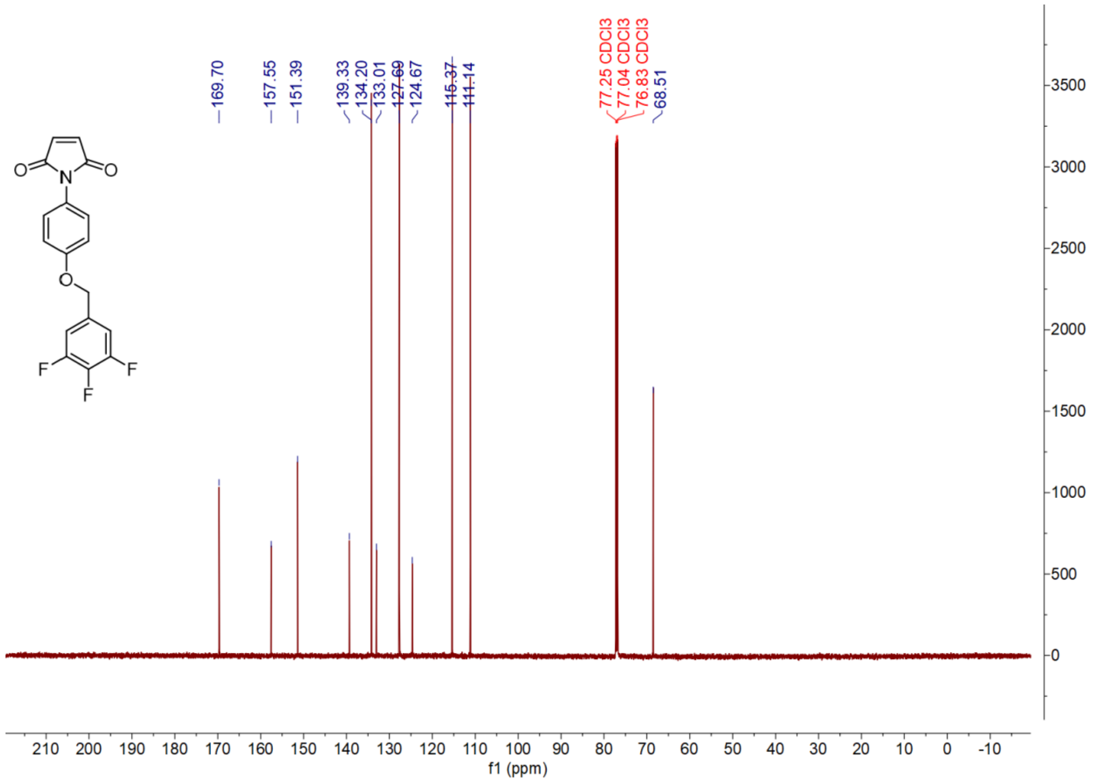

Fig. S14 ^{13}C NMR spectrum of M3F in $CDCl_3$.

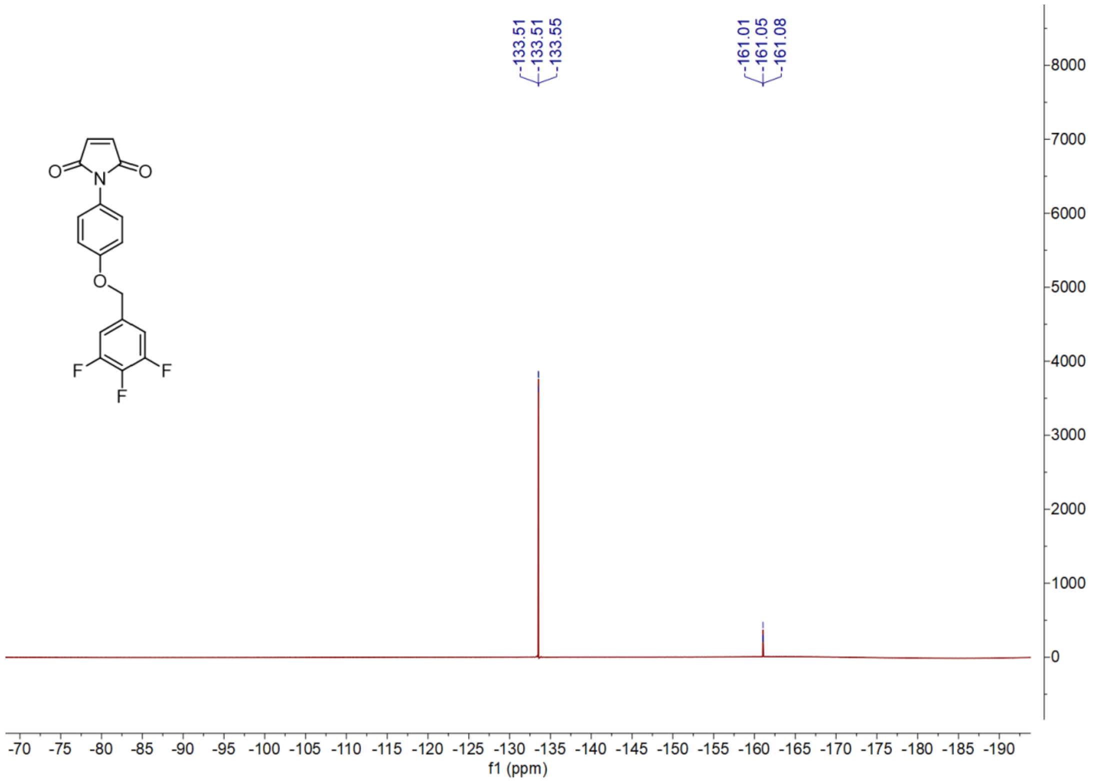

Fig. S15 ^{19}F NMR spectrum of M3F in $CDCl_3$.

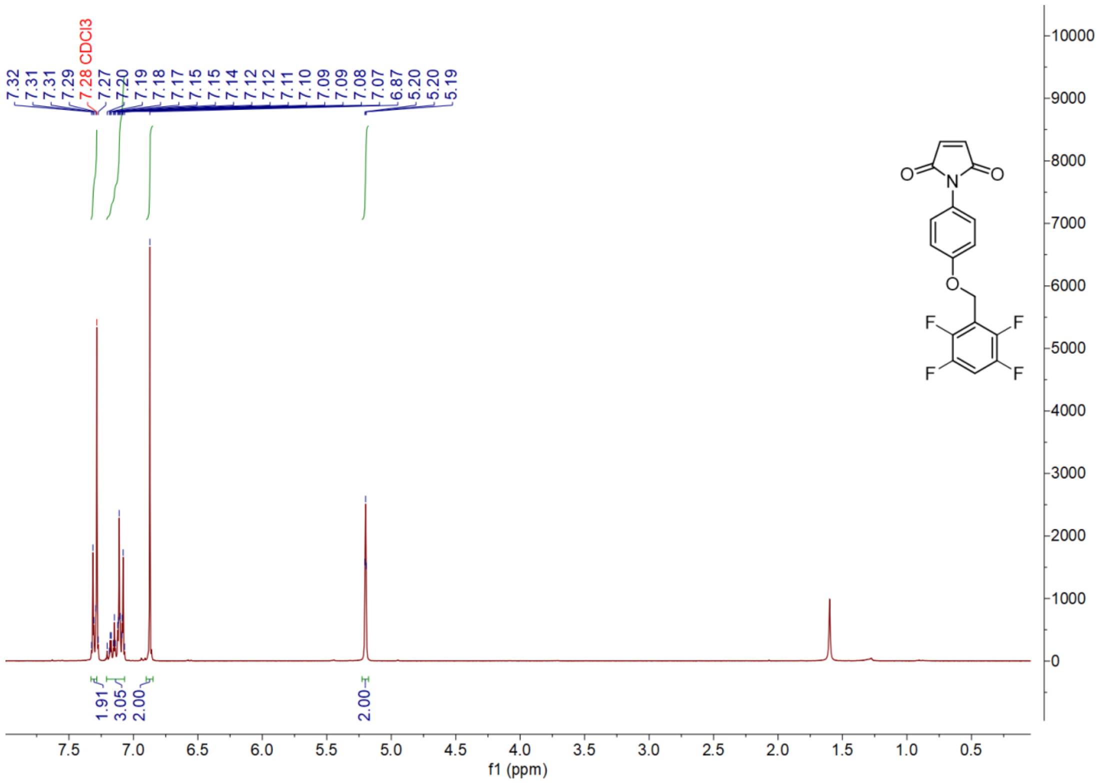

Fig. S16 ^{1}H NMR spectrum of M4F1 in $CDCl_3$.

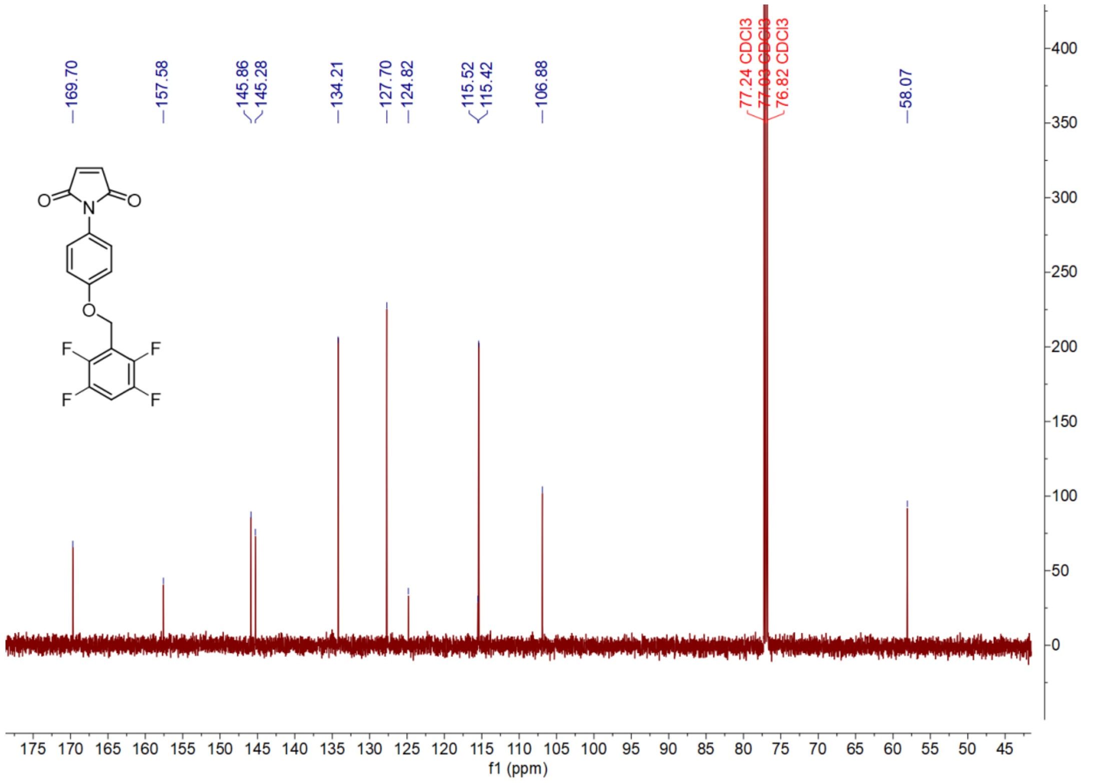

Fig. S17 ^{13}C NMR spectrum of M4F1 in $CDCl_3$.

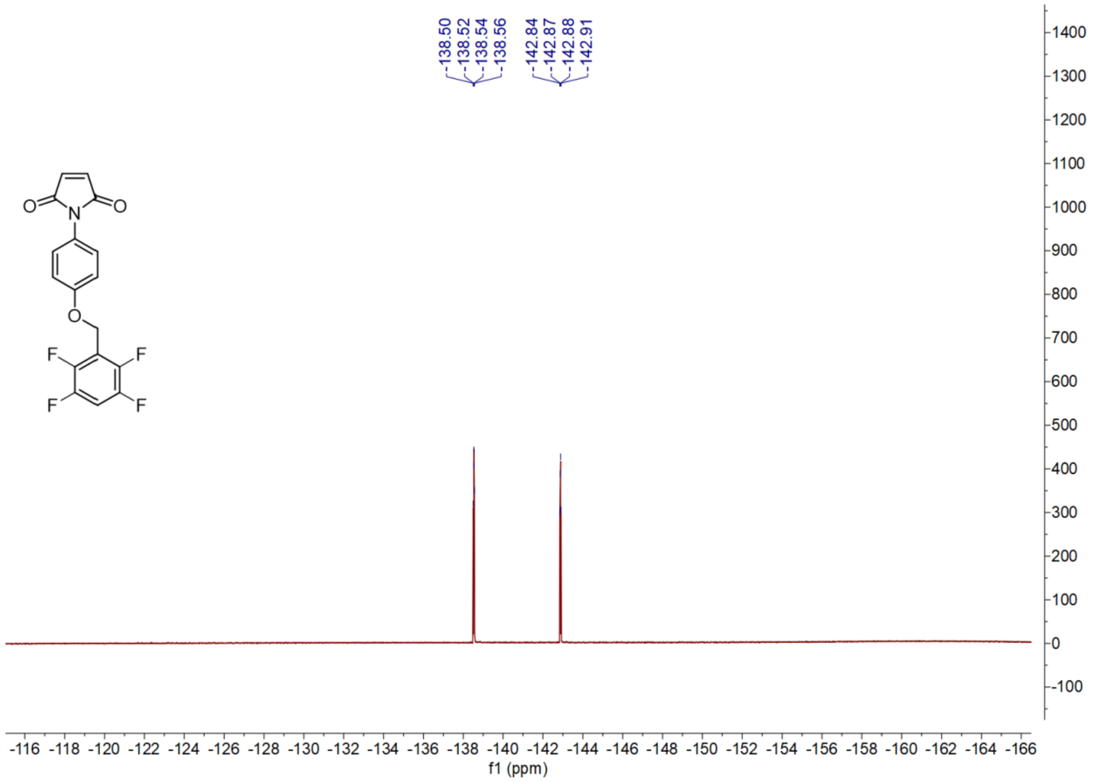

Fig. S18 ^{19}F NMR spectrum of M4F1 in $CDCl_3$.

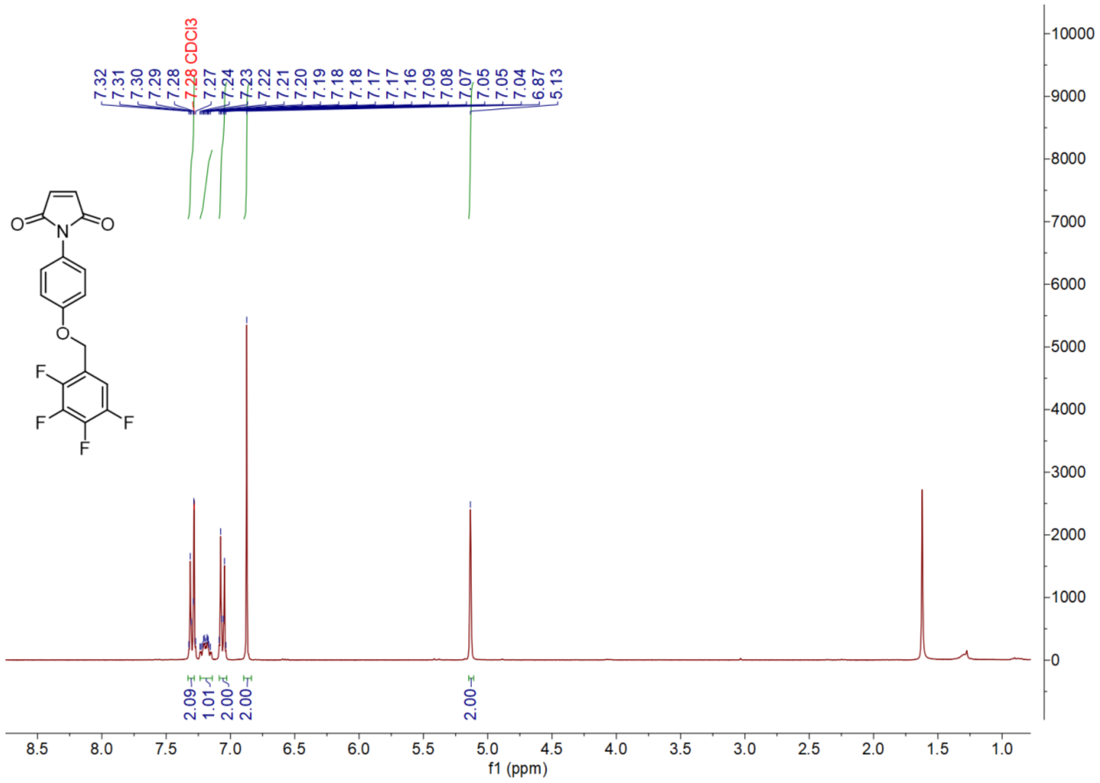

Fig. S19 ^{1}H NMR spectrum of M4F2 in $CDCl_3$.

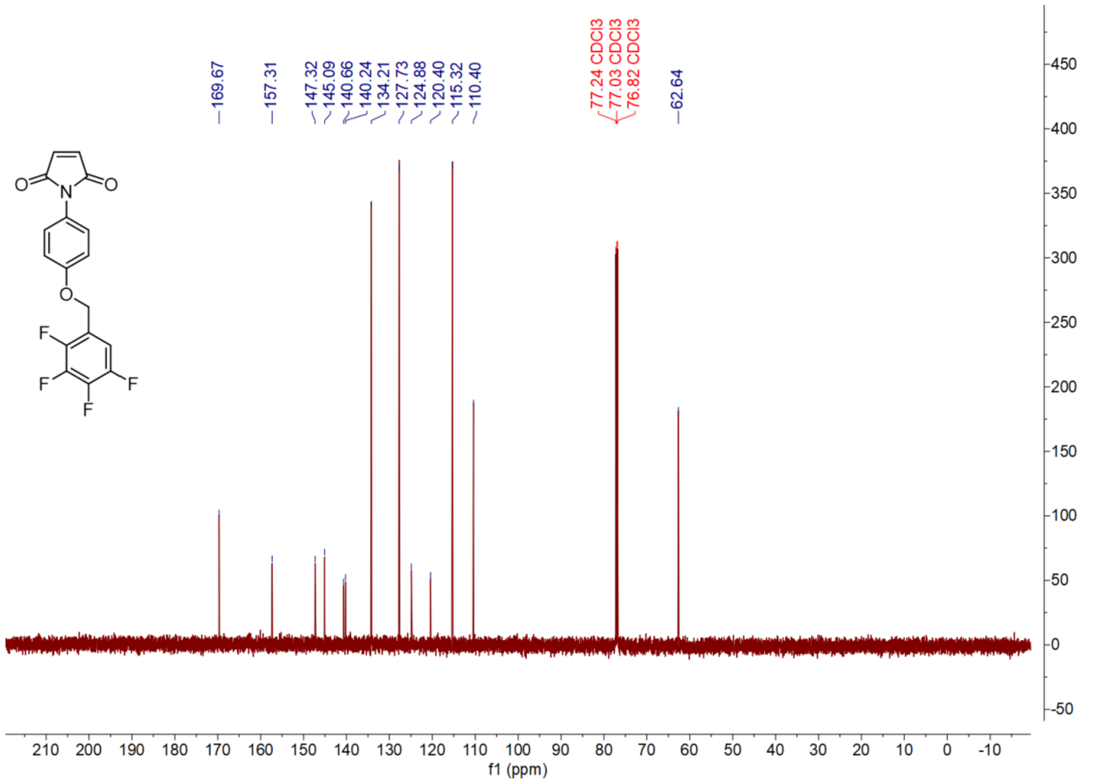

Fig. S20 ^{13}C NMR spectrum of M4F2 in $CDCl_3$.

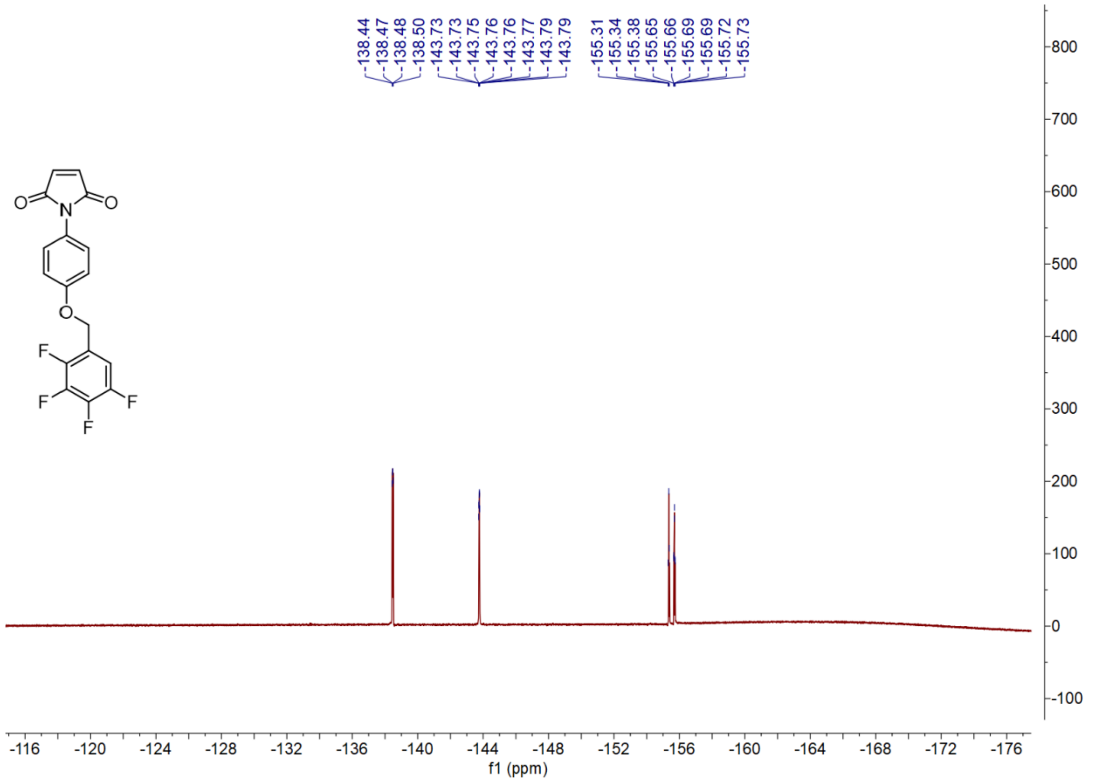

Fig. S21 ^{19}F NMR spectrum of M4F2 in $CDCl_3$.

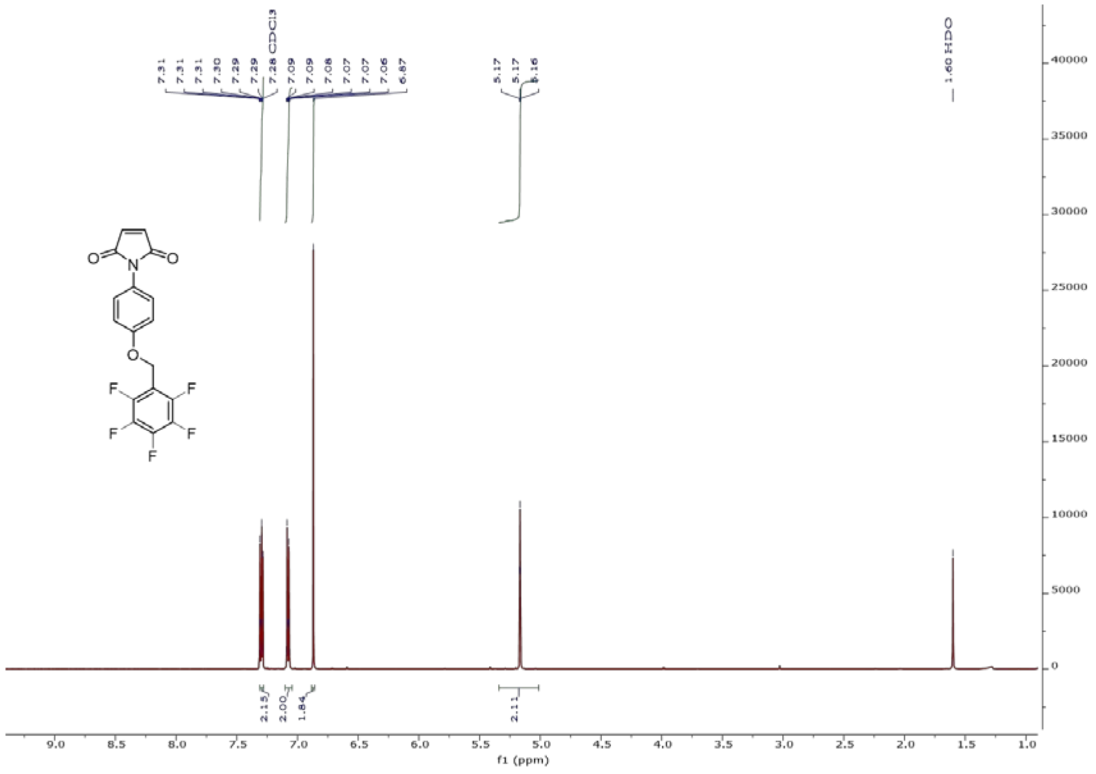

Fig. S22 ^{1}H NMR spectrum of M5F in $CDCl_3$.

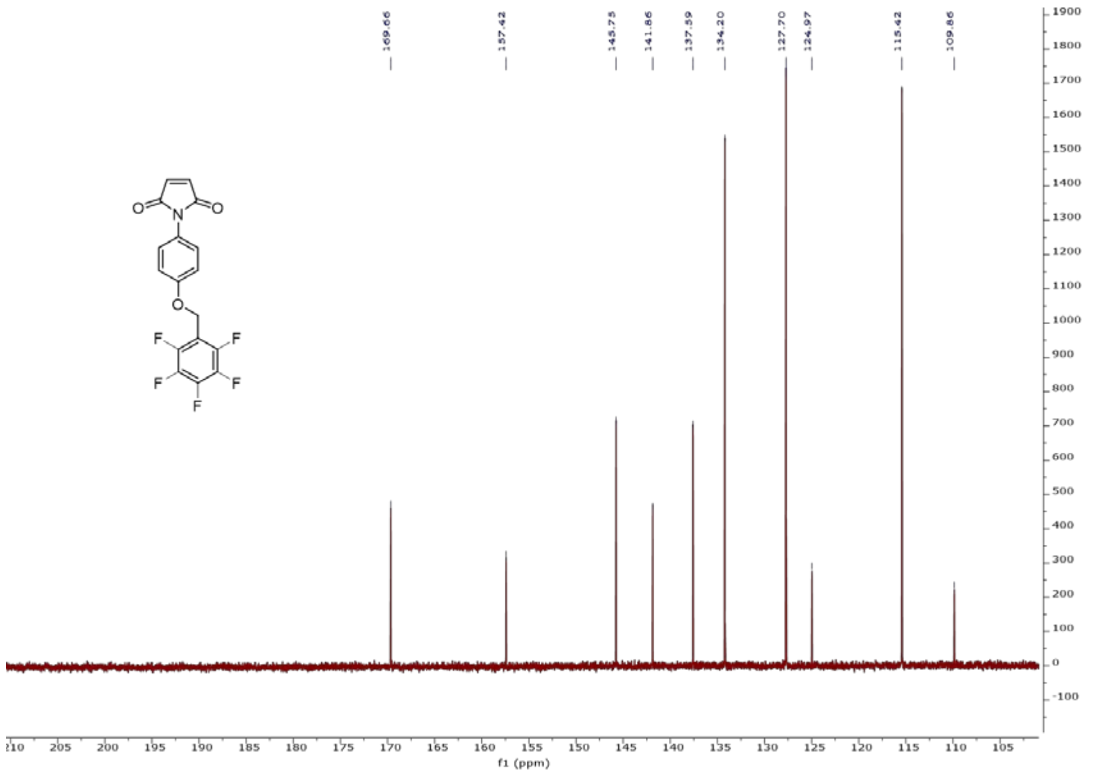

Fig. S23 ^{13}C NMR spectrum of M5F in $CDCl_3$.

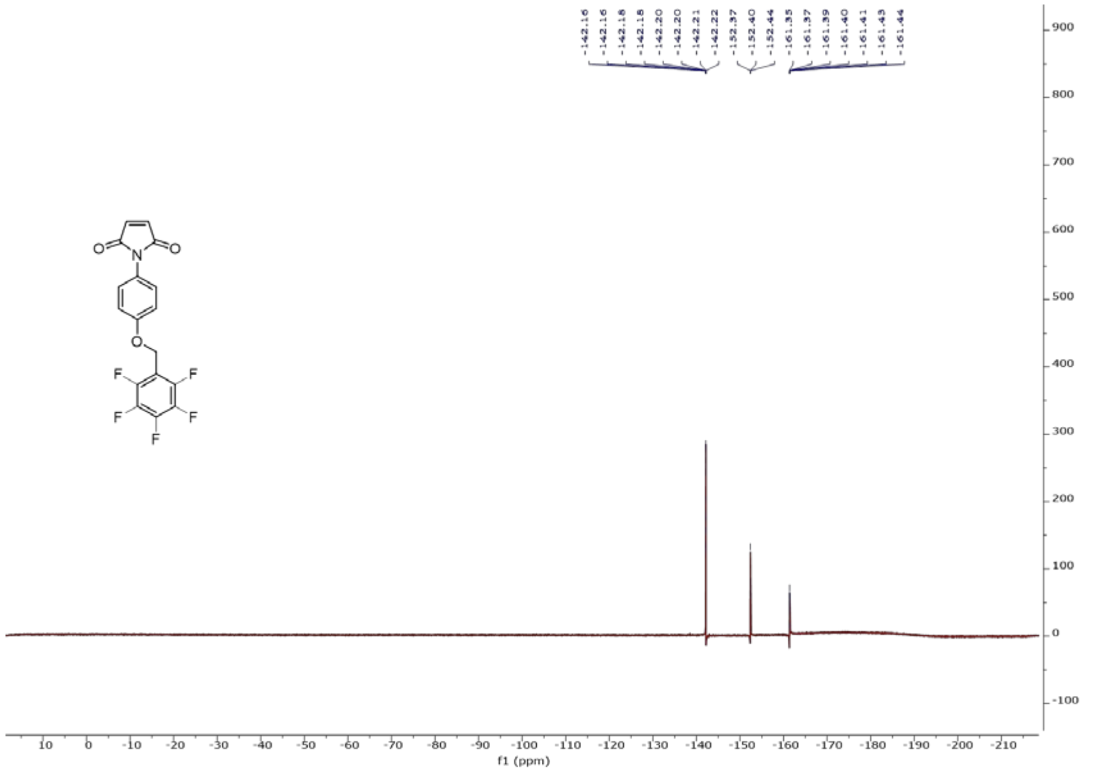

Fig. S24 ^{19}F NMR spectrum of M5F in $CDCl_3$.

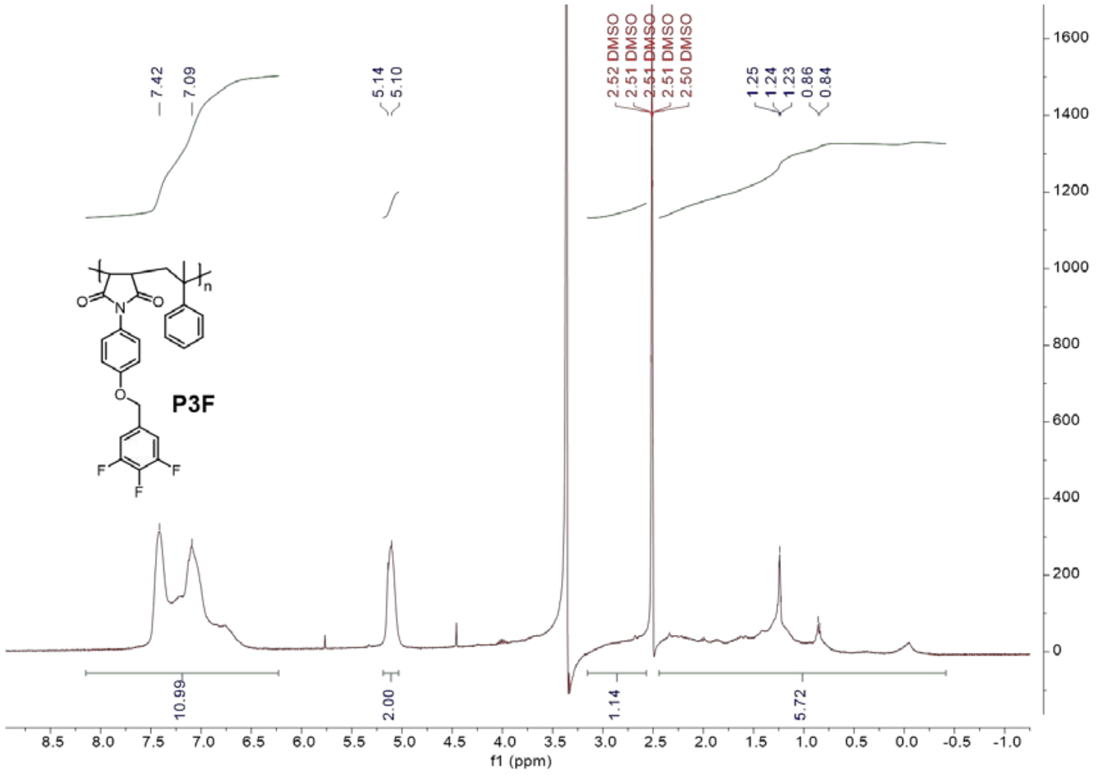

Fig. S25 ^{1}H NMR spectrum of P3F in DMSO.

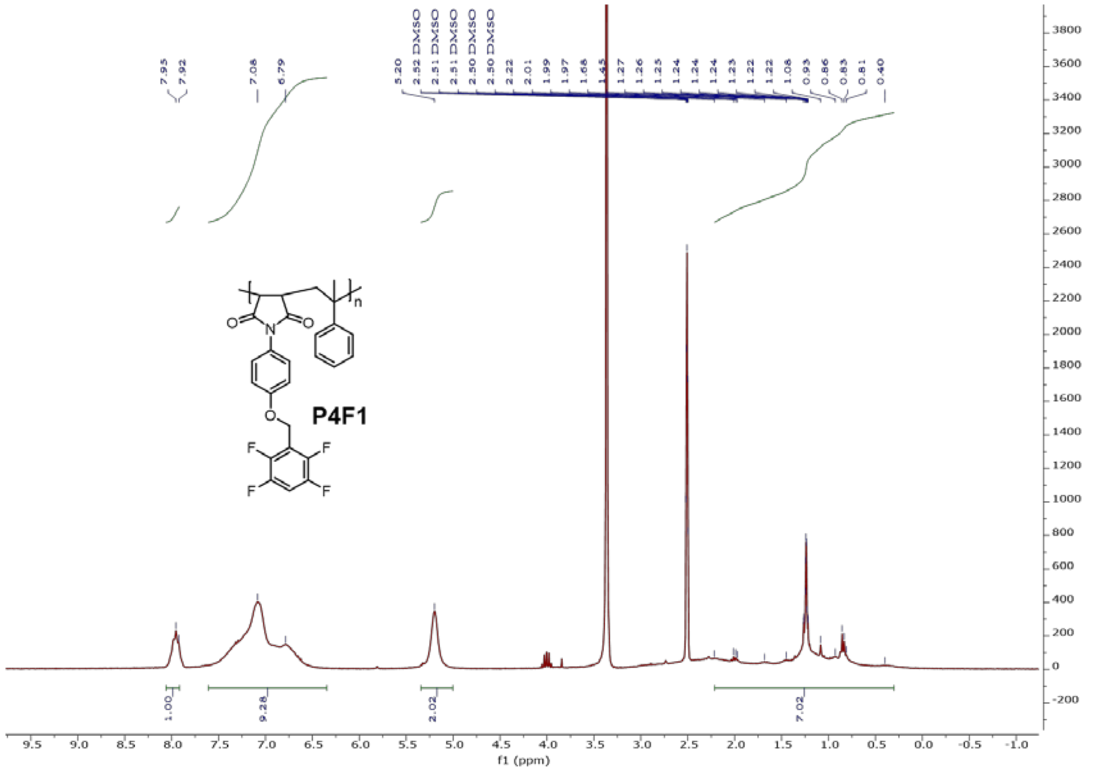

Fig. S26 ^{1}H NMR spectrum of P4F1 in DMSO.

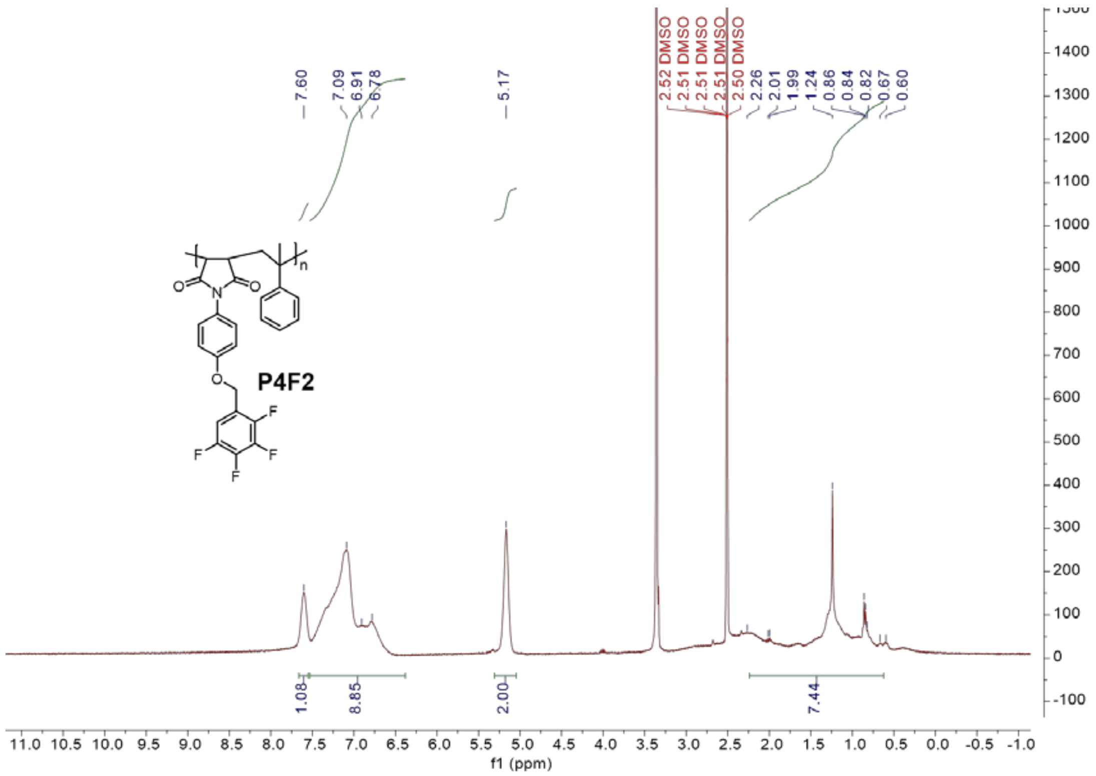

Fig. S27 ^{1}H NMR spectrum of P4F2 in DMSO.

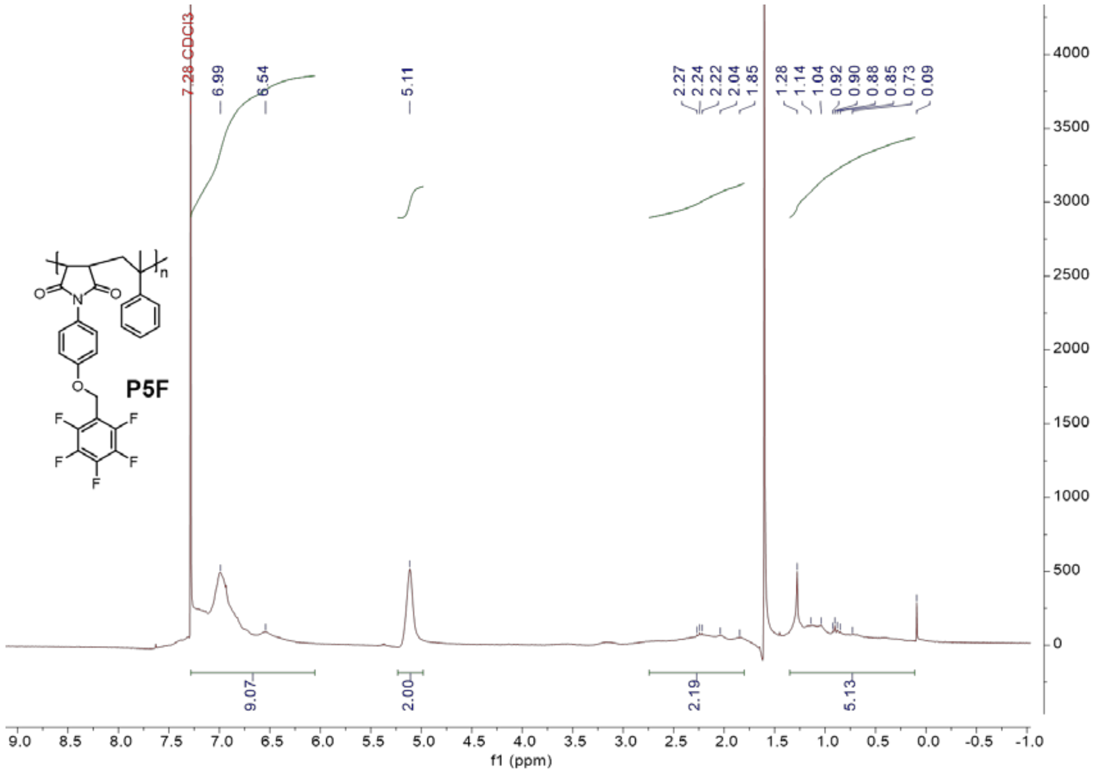

Fig. S28 ^{1}H NMR spectrum of P5F in $CDCl_3$.

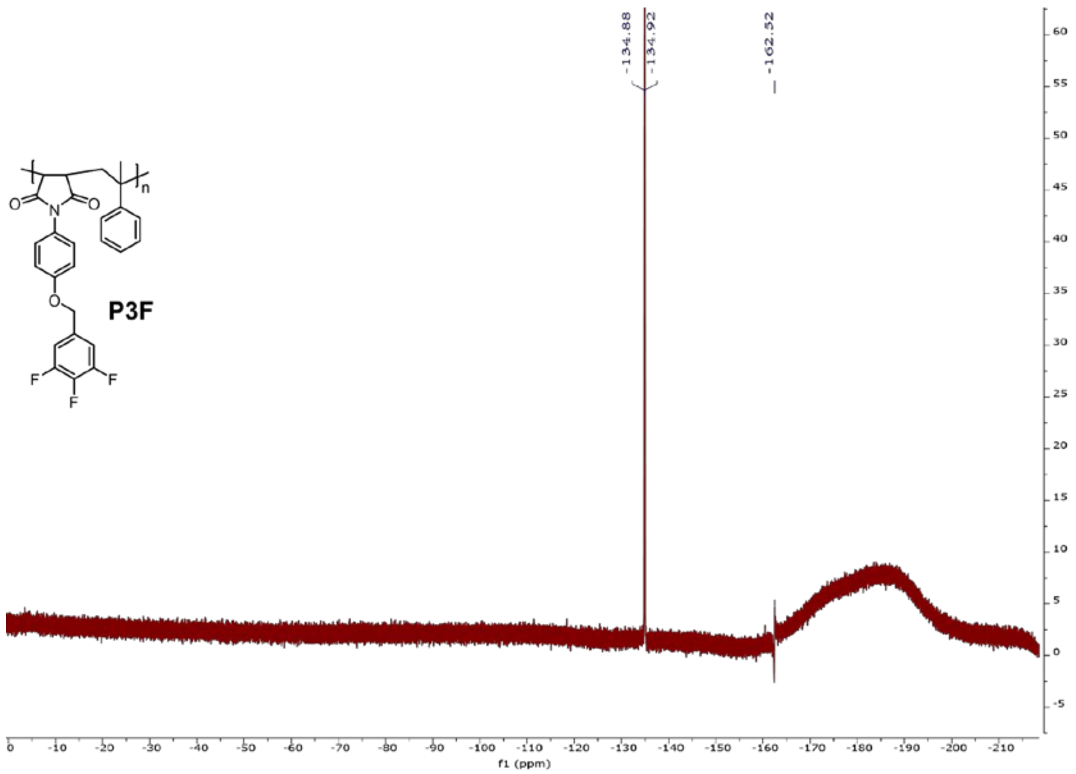

Fig. S29 ^{19}F NMR spectrum of P3F in DMSO.

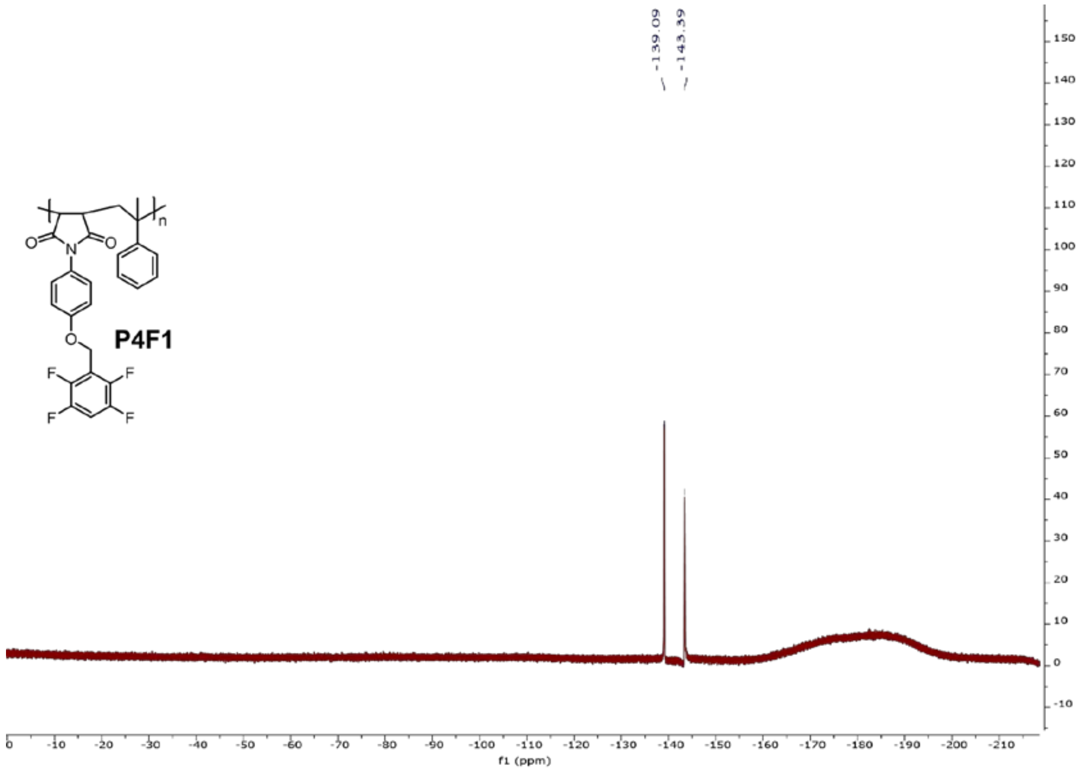

Fig. S30 ^{19}F NMR spectrum of P4F1 in DMSO.

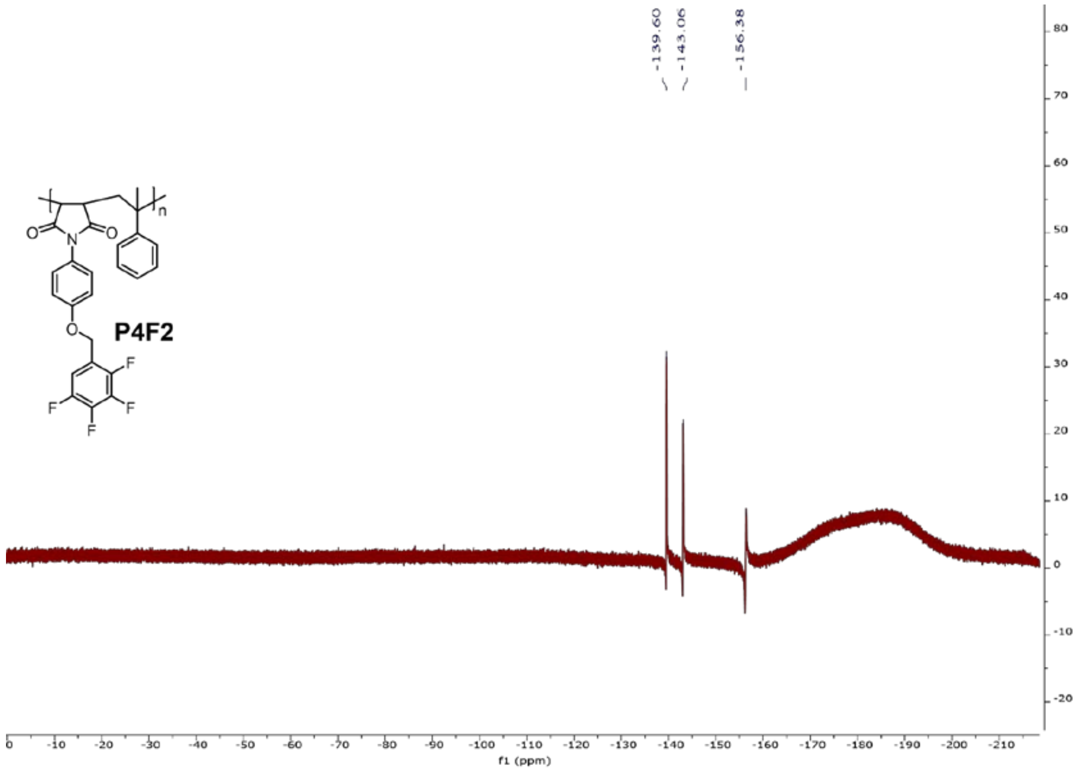

Fig. S31 ^{19}F NMR spectrum of P4F2 in DMSO.

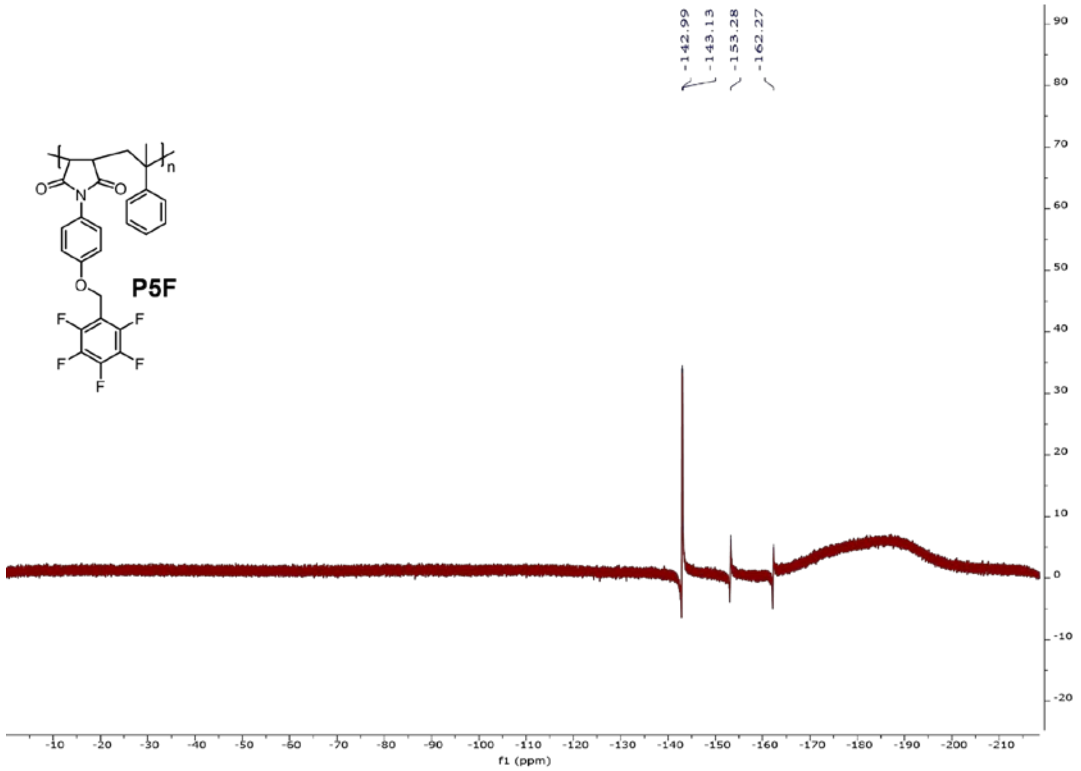

Fig. S32 ^{19}F NMR spectrum of P5F in DMSO.

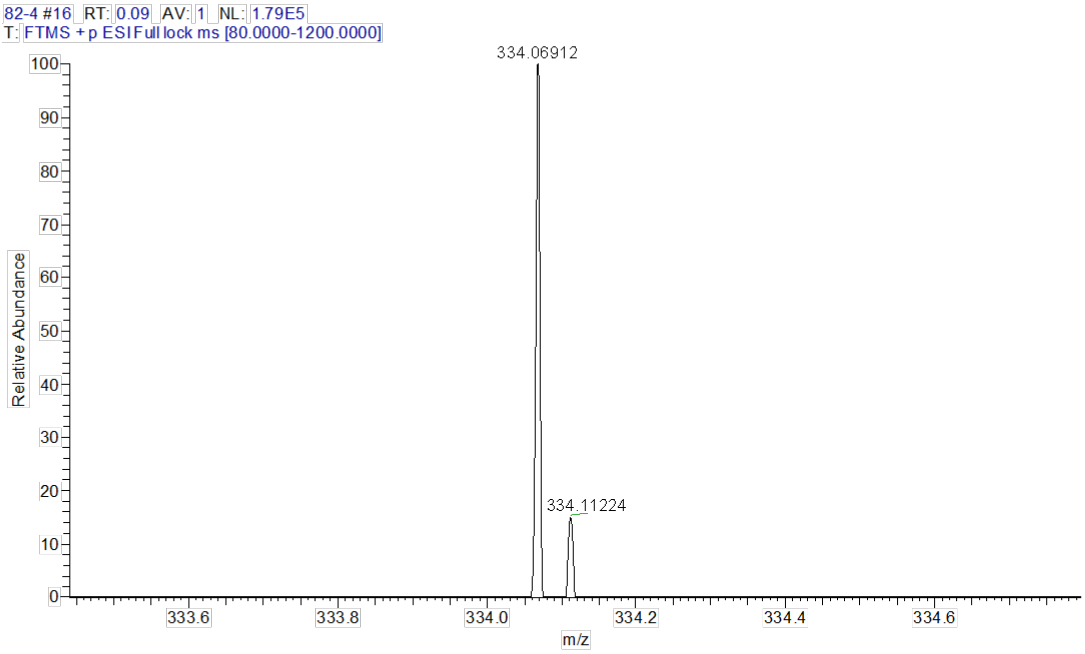

Fig. S33 HRMS spectrum of M3F in methanol.

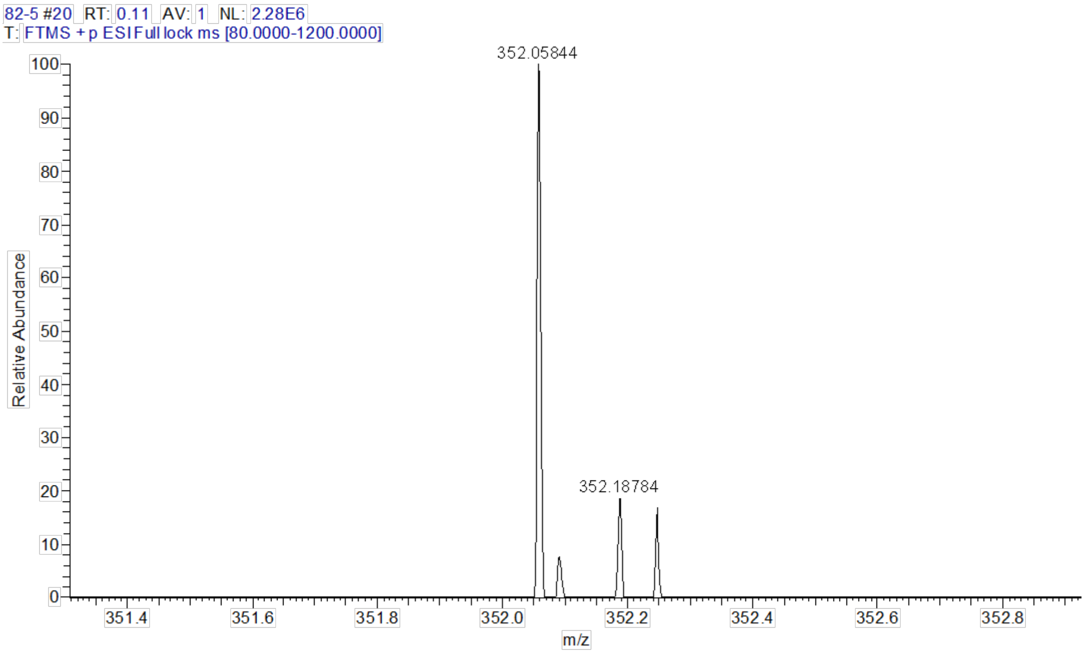

Fig. S34 HRMS spectrum of M4F1 in methanol.

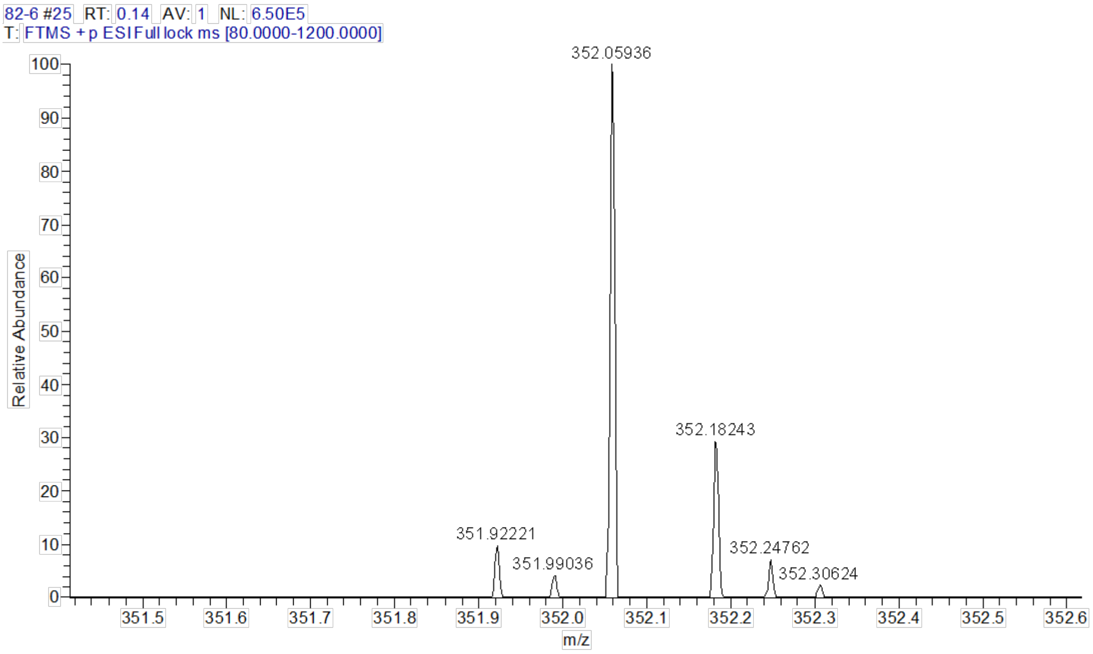

Fig. S35 HRMS spectrum of M4F2 in methanol.

X-ray data of the two maleimido monomers

Table S1. Data collection and summary of single-crystal experiment.

Chromophore	M4F1	M4F2
Empirical formula	C17H9F4NO3	C17H9F4NO3
Formula weight	351.25	351.25
Temperature [K]	243(2)	213(2)
Crystal system	orthorhombic	monoclinic
Space group (number)	P n a 21	P 1 21/n 1
a [Å]	31.7833(9)	18.7118(11)
b [Å]	5.3725(2)	3.8260(3)
c [Å]	8.4846(3)	21.4379(13)
α [°]	90	90
β [°]	90	111.612(4)
γ [°]	90	90
Volume [Å3]	1448.79(8)	1426.87(17)
Z	4	4
ρcalc [gcm−3]	1.61	1.635
μ [mm−1]	1.273	1.293
F(000)	712	712
2θ range [°]	5.56 to 145.43(0.81 Å)	5.373 to 147.3
Index ranges	−39 ≤ h ≤ 39	-22<=h<=23,
	−6 ≤ k ≤ 6	-4<=k<=3
	−10 ≤ l ≤ 10	-26<=l<=24
Reflections collected	22434	8855
Independent reflections	2842	2874
	Rint = 0.0786	R int= 0.0915
	Rsigma = 0.0525	
Coverage of independent reflections	99.9	98.1
Data / Restraints / Parameters	2842 / 325 / 289	2874 / 0 / 226
Goodness-of-fit on F2	1.087	1.056
Final R indexes	R1 = 0.0571	R1 = 0.1061
[I≥2σ(I)]	wR2 = 0.1557	wR2 = 0.2443
Final R indexes	R1 = 0.0621	R1 = 0.1294
[all data]	wR2 = 0.1647	wR2 = 0.2779
Largest peak/hole [eÅ−3]	0.23/−0.32	0.554 /-0.526

Molecular weight characterization of polymers

Table S2. The molecular weights and polydispersity indices (Đ) of the polymers were characterized by gel permeation chromatography (GPC) using a system operated at room temperature with N, N-dimethylformamide (DMF) as the eluent.

Polymer	$\overline{M}_w$ (Da.)	Đ	T_g (℃)
P3F	8835	2.2	112
P4F1	29714	2.7	182
P4F2	18386	2.3	137
P5F	31600	2.9	160

EO analyses under high-frequency and low-frequency modulation voltages

Table S3. A comparison of 6-Hz and 600-Hz data was conducted for the three systems: **P4F1/AJLZ53**-35%, **P5F1/AJLZ53**-35%, and **PC/AJLZ53**-35%.

	Frequency	**P4F1/AJLZ53**-35%	**P5F/AJLZ53**-35%	**PC/AJLZ53**-35%
r_{13}/r_{33} at 1306 nm (pm V^{-1})	600 Hz	51.4/161.9	47.1/144.7	57.0/160.0
	6 Hz	51.9/177.5	46.6/159.0	56.0/169.2
r_{13}/r_{33} at 1541 nm (pm V^{-1})	600 Hz	39.6/102.8	35.6/100.9	39.2/104.0
	6 Hz	37.0/101.7	32.9/93.8	38.0/106.5

Summary of methods for calculating EO coefficients from ATR technique

$$\Delta n_{\mathrm{TE}} = -\frac{n_{\mathrm{TE}}^3 r_{13} E_{\mathrm{mod}}}{2} \quad \text{(Equation S1)}$$

$$\Delta n_{\mathrm{TM}} = -\frac{n_{\mathrm{TM}}^3 r_{33} E_{\mathrm{mod}}}{2} \quad \text{(Equation S2)}$$

$$E_{\mathrm{mod}} = \frac{V_{\mathrm{mod}}}{d} \quad \text{(Equation S3)}$$

$$\Delta R_{\mathrm{TE}} = \frac{\partial R_{\mathrm{TE}}}{\partial N_{\mathrm{eff}}^s}\frac{\partial N_{\mathrm{eff}}^s}{\partial n_{\mathrm{TE}}}\Delta n_{\mathrm{TE}} = \frac{\partial R_{\mathrm{TE}}}{\partial N_{\mathrm{eff}}^s}\Delta n_{\mathrm{TE}} \quad \text{(Equation S4)}$$

$$\Delta R_{\mathrm{TM}} = \frac{\partial R_{\mathrm{TM}}}{\partial N_{\mathrm{eff}}^p}\frac{\partial N_{\mathrm{eff}}^p}{\partial n_{\mathrm{TE}}}\Delta n_{\mathrm{TE}} + \frac{\partial R_{\mathrm{TM}}}{\partial N_{\mathrm{eff}}^p}\frac{\partial N_{\mathrm{eff}}^p}{\partial n_{\mathrm{TM}}}\Delta n_{\mathrm{TM}} \quad \text{(Equation S5)}$$

$$r_{13} = \frac{2d}{n_{\mathrm{TE}}^3 V_{\mathrm{mod}}}\Delta n_{\mathrm{TE}} = \frac{2d}{n_{\mathrm{TE}}^3 V_{\mathrm{mod}}}\Delta R_{\mathrm{TE}}/(\frac{\partial R_{\mathrm{TE}}}{\partial N_{\mathrm{eff}}^s}\frac{\partial N_{\mathrm{eff}}^s}{\partial n_{\mathrm{TE}}}) \quad \text{(Equation S6)}$$

$$r_{33} = \frac{2d}{n_{\mathrm{TM}}^3 V_{\mathrm{mod}}}[\Delta R_{\mathrm{TM}} + \frac{\partial R_{\mathrm{TM}}}{\partial N_{\mathrm{eff}}^p}\frac{\partial N_{\mathrm{eff}}^p}{\partial n_{\mathrm{TE}}}\frac{n_{\mathrm{TE}}^3 r_{13} V_{\mathrm{mod}}}{2d}]/(\frac{\partial R_{\mathrm{TM}}}{\partial N_{\mathrm{eff}}^p}\frac{\partial N_{\mathrm{eff}}^p}{\partial n_{\mathrm{TM}}}) \quad \text{(Equation S7)}$$

$$r_{13} = 2dN_{\mathrm{eff}}\sqrt{2\Delta R_{\mathrm{TE}}/\left(\frac{\partial^2 R_{\mathrm{TE}}}{\partial {N_{\mathrm{eff}}^s}^2}\right)}\Big/(n_{\mathrm{TE}}^4 V_{\mathrm{mod}}) \quad \text{(Equation S8)}$$

$$r_{33} = 2d\sqrt{2\Delta R_{\mathrm{TM}}/\left(\frac{\partial^2 R_{\mathrm{TM}}}{\partial {N_{\mathrm{eff}}^p}^2}\right)}\Big/(n_{\mathrm{TM}}^2 N_{\mathrm{eff}} V_{\mathrm{mod}}) \quad \text{(Equation S9)}$$

$$r_{13} = \frac{2d\Delta n_{\mathrm{TE}}}{n_{\mathrm{TE}}^3 V_{\mathrm{mod}}} = \frac{d}{n_{\mathrm{TE}}^3}\left(\frac{2\Delta N_{\mathrm{eff}}}{V_{\mathrm{mod}}}\right) \quad \text{(Equation S10)}$$

$$r_{33} = \frac{2d\Delta n_{\mathrm{TM}}}{n_{\mathrm{TM}}^3 V_{\mathrm{mod}}} = \frac{d}{n_{\mathrm{TM}}^3}\left(\frac{2\Delta N_{\mathrm{eff}}}{V_{\mathrm{mod}}}\right) \quad \text{(Equation S11)}$$

Parameters	Description
n_{TE}, n_{TM}	Anisotropic refractive indices of the poled film for TE and TM polarizations, respectively.
d	Thickness of the poled polymer film.
V_{mod}	Amplitude of the applied modulation voltage.
E_{mod}	Strength of the applied modulation electric field.
Δn_{TE}, Δn_{TM}	Pockels effect-induced change in the refractive index along the TE and TM direction, respectively..
r_{13}, r_{33}	Components of the linear EO coefficient tensor.
R_{TE}, R_{TM}	ATR reflectivity spectrum for the TE and TM mode, respectively.
ΔR_{TE}, ΔR_{TM}	Experimentally measured change in reflectivity for the Transverse Electric (TE) and Transverse Magnetic (TM) mode, respectively.
N_{eff}^s, N_{eff}^p	Effective refractive index for *s*-polarized (TE) and *p*-polarized (TM) light from ATR spectra, respectively.

Supplementary Note for the calculation of EO coefficients[1,2]

1. Equations S6 and S7 are the same as Equations 1 and 2 in the main texts, respectively, by applying a first-order Taylor expansion as a linear approximation.
2. Equations S8 an S9 use the second-order term of Taylor expansion and assume the electric field induces a ΔN_{eff} translation of ATR spectra without altering the shape of the minimum peak, applicable to broad modes by the high-frequency lock-in-free ATR measurements beyond the Nyquist limit (Table S3, TM modes).
3. For EO modulation that induces significant mode splitting, the *r*-coefficients are directly calculated by Equations S10 and S11 (Table S3, TE modes).

Calculation of order parameters of poled films from the poling-induced optical birefringences

A Sellmeier fit of refractive index is carried out based on refractive index at 1.3 µm and 1.54 µm, where the refractive index (n) is contributed by non-resonant background (n_0) and the two-level charge transfer resonance at frequency v_0 in cm^{-1}.[3,4]

$$n = n_0 + \frac{A}{v_0^2 - v^2}$$

where n and v are the refractive index of unpoled films and frequency at 1.3 µm and 1.54 µm, respectively. The v_0 is the frequency of chromophore/polymer films at the maximum absorption wavelength. The order parameter (Φ) for poled films is given by

$$\Phi = \frac{\delta_{TM}-\delta_{TE}}{\delta_{TM}+2\delta_{TE}}$$

Where $\delta_{TM} = n_{TM}-n_0$ and $\delta_{TE} = n_{TE}-n_0$.

The n_{TM} and n_{TE} are the experimentally measured refractive indices of poled films in transverse magnetic (TM) and transverse electric (TE) polarization, respectively.

References:

[1] Lyu, D.; Zhang, D.; Luo, J. Lock-in free ATR detections of organic thin film pockels effects within and beyond Nyquist. *ACS Photonics* **2024**, *11*, 1780–1792.

[2] Lyu, D.; Zhang, D.; Luo, J. Accurate, multimode, and lock-in free ATR detection of benchmark electro-optical materials. *ACS Appl. Opt. Mater.* **2025**, *3*, 1637–1650.

[3] Page, R. H.; Jurich, M. C.; Reck, B.; Sen, A.; Twieg, R. J.; Swalen, J. D.; Bjorklund, G. C.; Willson, C. G. Electrochromic and optical waveguide studies of corona-poled electro-optic polymer films. *J. Opt. Soc. Am. B*, **1990**, 7, 1239-1250.

[4] Burland, D. M.; Miller, R. D.; Walsh, C. A. Second-order nonlinearity in poled-polymer systems. *Chem. Rev.*, **1994**, *94*, 31-75.
